# Reach: Recognising episodes of acute complexity in health: a predictive older patient prioritisation machine learning model

**DOI:** 10.1007/s10729-026-09783-5

**Published:** 2026-07-29

**Authors:** Abtin Ijadi Maghsoodi, Valery Pavlov, Paul Rouse, Cameron Graham Walker, Matthew Parsons

**Affiliations:** 1 Department of Information Systems and Operations Management, Faculty of Business and Economics, University of Auckland, Auckland, 1010 New Zealand; 2 Health New Zealand (Te Whatu Ora) – Waikato District, Hamilton, 3240 New Zealand; 3 Department of Accounting and Finance, Faculty of Business and Economics, University of Auckland, Auckland, 1010 New Zealand; 4 Department of Engineering Science, Faculty of Engineering, University of Auckland, Auckland, 1010 New Zealand; 5 Division of Health, Engineering, Computing and Science, School of Health, University of Waikato, Hamilton, New Zealand

**Keywords:** Older person care, Geriatrics, Patient prioritisation, Machine learning, Classification, Pathway modelling

## Abstract

As the numbers of older people (65 +) rise globally, the pressure on acute hospitals to provide efficient and effective care while addressing resource inequities increases. In this study we introduce Recognising Episodes of Acute Complexity in Health (REACH), a novel Automatic Machine Learning (AutoML)-based predictive model that prioritises older patients and assigns them to complex or non-complex care pathways using routinely collected electronic health record (EHR) and interRAI data. Existing triage and patient-flow approaches are rarely designed for equity-sensitive deployment in settings where underserved populations experience systematically different access and outcomes; in our context, this gap motivated the development of REACH. Designed to be flexible and adaptable, REACH was developed and stress-tested in New Zealand, where Māori, Pasifika and other underserved older populations face inequitable access and outcomes, and is intended as a transferable approach for other health systems with similar equity and capacity challenges. The model estimates individual complexity probabilities and incorporates resource and capacity constraints to support decisions about patient prioritisation, bed allocation and placement into older-person-specific pathways. Applied to seven years of hospital data, REACH demonstrates improved identification of complex geriatric cases and more efficient use of specialised ward capacity compared with traditional FIFO and triage-based assignment. Using New Zealand data as a real-world case study, we demonstrate that equity-aware, capacity-constrained pathway assignment can be operationalised with routine EHR/interRAI inputs, with a design that can be retrained and recalibrated for other jurisdictions that use comparable assessment standards. The application of the model demonstrates its potential to improve patient prioritisation and placement processes while addressing challenges in healthcare access for underserved populations, highlighting the importance of data-driven decision support systems in older persons' care. The findings hold significant implications for healthcare practitioners, managers, policymakers and researchers seeking to design equitable and efficient models of acute care for older people.

**Supplementary Information:**

The online version contains supplementary material available at https://doi.org/10.1007/s10729-026-09783-5.

## Highlights

- Developed REACH, an AutoML model for prioritising geriatric patient care.
- Tailored REACH for underserved populations in New Zealand's healthcare system.
- Improved patient flow via machine learning for complex and non-complex pathways.
- Leveraged EHR data for geriatric patient prioritisation and allocation.
- Enhanced equity in care with culturally informed patient prioritisation strategies.

## Introduction

As global life expectancy rises, healthcare systems, including that of New Zealand (NZ), are under growing pressure from an ageing demographic [1]. By 2023, NZ's population aged 65 and over will number approximately 842,000, projected to reach one million by 2028 [2]. However, these figures mask significant disparities within the population, particularly for Māori, who face greater barriers to accessing healthcare and experience poorer outcomes compared to non-Māori populations. In regions like the Waikato District, where the proportion of Māori is higher than the national average, these inequities are particularly evident. Māori often experience accelerated ageing and earlier onset of comorbidities, making the typical threshold of 65 years an inadequate indicator of their health needs. This demographic shift and its disparities present challenges in managing comorbidity among older people, especially underserved groups, intensifying the need for prioritised treatment strategies [3]. However, existing triage and allocation approaches are not designed to systematically identify complex geriatric patients or to optimise the use of specialised older-persons capacity in a transparent, equity-focused way.

These challenges are further exacerbated for underserved populations with limited access to timely care as prolonged wait times increase morbidity, mortality, and readmissions [4]. Effectively managing and prioritising patient care is essential to mitigate these adverse effects and ensure that those with the most severe needs receive timely treatment [5, 6]. However, healthcare providers increasingly struggle to provide appropriate care for frail and acutely ill older patients. This research explores approaches for delivering appropriate care to these patients by introducing a patient prioritisation and pathway assignment tool.

Recent studies underscore the importance of early patient assignment in Emergency Departments (ED) as crucial to effective triaging, rather than waiting until the ward assignment stage [7–12]. This strategy aligns with best practices in emergency response, enhancing patient satisfaction, reducing length-of-stay and improving education for residents [13]. Addressing these issues requires a holistic approach that incorporates both individual patient needs and broader resource management strategies, including equitable care allocation for underserved groups such as older adults facing complex health challenges[14]. Addressing these issues requires a holistic approach that incorporates both individual patient needs and broader resource management strategies, such as targeted care initiatives for older patients[14]. In acute care settings, older patients predominantly occupy hospital beds and face higher rates of adverse outcomes, such as in-hospital mortality and prolonged stays associated with polypharmacy, sarcopenia, pressure ulcers, pneumonia and other life-threatening events such as sepsis and opioid overdose [4]. For underserved populations, these challenges are compounded by systemic barriers, such as limited access to preventative care and geographic isolation, which further strain healthcare resources. To address these challenges, older-person-specific care models such as Acute Care for Elders (ACE) and Geriatric Evaluation and Management Units (GEMU) have been developed to enhance health outcomes and reduce hospital stays and healthcare costs [15, 16]. While primary and secondary patient outcomes have been widely reported, the literature on improving older patients’ journeys based on their appropriate resource allocation and pathway prioritisation remains limited.

Healthcare organisations are required to balance cost savings and service improvement, despite capacity constraints and patient demand fluctuations [4, 17]. Service providers often face a supply–demand mismatch, resulting in overcrowding, delays, staffing issues and dissatisfaction among staff and patients. Addressing this mismatch is vital for reducing patient flow problems. Prioritising hospital patient flow management is crucial for maintaining equilibrium between supply and demand [18]. This study tracks patient movements through the care process and critically examines strategies for assigning patients to appropriate care pathways and prioritising their treatment plans.

EDs are dynamic and complex environments where patients with diverse and unpredictable health issues arrive [19]. A triage system is essential to categorise patients based on urgency, rapidly sorting incoming patients to appropriate pathways by the severity of their illness [20] and anticipated operational needs [21]. A common method to assign patients to suitable pathways involves triage tools. The Emergency Severity Index (ESI) is an example that assesses patient acuity, considering urgency, severity and resource needs to gauge case complexity [22]. Once assigned at triage, it ranks patients from most to least severe. To reduce wait times for specific priority levels, a widely adopted strategy known as Fast Track is employed [23]. This strategy proactively dedicates specific capacity to efficiently allocate patients, thereby reducing delays and adverse patient outcomes. For Māori, Pacifica and other underserved populations, culturally tailored adaptations of models such as Fast Track (FT) are essential to ensure equitable access to care and mitigate existing disparities. ACE serves as an adaptation of FT, concentrating on those with higher acuity.

Triage regulates patient flow in overcrowded environments [24] but triage systems sometimes lack clear guidelines for routing patients within urgency levels, leaving health professionals to rely on their judgment [25]. In contrast, patient prioritisation tools help rank inpatient and outpatient referrals by various criteria, enabling equitable access to care and enhancing waitlist management systems, particularly for underserved populations [26]. Unlike First-In, First-Out (FIFO) methods that arrange patients chronologically, prioritisation balances clinical needs within a broader health strategy. While some studies suggest comparing patient prioritisation to triage methods [27], others argue that prioritisation is more comprehensive, balancing various clinical needs within a holistic health strategy that extends beyond urgent care[28]. Conventional emergency department triage systems therefore provide point-in-time acuity categories based mainly on presenting signs and symptoms, with limited incorporation of longitudinal EHR or interRAI information, no explicit modelling of downstream bed capacity or ward configuration, and little support for equity-focused prioritisation of complex geriatric patients. REACH is motivated by these limitations and is designed as a complementary decision-support tool that augments, rather than replaces, triage by focusing on complexity and pathway assignment over the whole admission episode.

In order for EDs and the wider hospital to remain effective, wait times must be minimised and length-of-stay and resource use must be optimal, but without impacting the effectiveness of other departments; a challenge due to the higher resource needs of older patients. Initially, this study focused on establishing older-persons-specific FT the Older Persons and Rehabilitation (OPR) ward at Health New Zealand (HNZ) Waikato District. This was later expanded to incorporate: (a) improved patient assignment to appropriate pathways, including triaging patients and prioritising complex and non-complex pathways; and (b) logically segregating resources and allocating them across prioritised patient streams within a department, which can enhance system performance through more effective partitioning of capacity.

Research using Artificial Intelligence (AI) and Machine Learning (ML) techniques has addressed patient flow management, focusing on bed capacity and overcrowding [29–31]. However, optimal pathways for older patients are underexplored, particularly in formulating suitable prioritisation strategies. This study explores how patients can be assigned to appropriate pathways, considering the patient flow for complex and non-complex geriatric patients. The proposed solution has been tailored to HNZ Waikato District to handle complex versus non-complex patient categories, ensuring tailored and efficient care pathways. A predictive ML model was developed using a combination of gradient boosting architectures and regression tree models, assembled through an AutoML framework, to facilitate patient allocation to pathways tailored for complex and non-complex cases. This model integrates existing configurations, such as merging wards and units to establish a specialised Fast Track. Although decision support tools for patient prioritisation exist, this is the first study in NZ to apply actual patient pathway assignment settings with historical Electronic Health Records (EHR), incorporating demographic and equity considerations alongside the international resident assessment instrument (interRAI). This study integrates older-person-specific models such as ACE into the Fast Track pathway, focusing on complex and non-complex patient categories.

Prediction models for patient prioritisation are often developed using discharge data; however, focusing on high-risk patients only at discharge is largely ineffective, as it misses the critical window for meaningful intervention [18, 32]. By the time of discharge, opportunities to prevent readmissions or significantly improve outcomes have already passed. Instead, early identification during hospitalisation is essential, and this is made possible through the detailed and comprehensive data available in EHRs supplemented with the between-admissions data.

This study introduces the Recognising Episodes of Acute Complexity in Health (REACH), a predictive tool for geriatric patient pathway assignment and prioritisation, demonstrating its potential superiority over traditional FIFO and triaging models by jointly modelling patient complexity and ward capacity when assigning patients to complex versus non-complex pathways. Combining alternative pathways with ward aggregation results in significant performance improvement, mediated by several factors affecting process focus. In addition to developing a general patient prioritisation and pathway assignment tool, considering factors such as Patient Clinical Complexity Level (PCCL), Australian Refined Diagnosis Related Groups (AR-DRG) and International Classification of Diseases (ICD), a customised prioritisation tool was also created for acute older patients using the interRAI clinical assessment instrument. This tool thus enhances care continuity, supports a person-centred approach and boosts the system’s capacity to assess clinical outcomes.

## Literature review

In recent years, significant advances have been made in the application of operations research/management (OR/OM) tools [23] and clinical developments [32], to the patient journey through tailored, patient-centred prioritisation and triaging tools. Studies on patient pathway assignment, prioritisation and triage; including both medical and non-medical solutions, are particularly relevant to the current study.

While closely aligned with the extensive literature on patient flow and pathway analysis, our focus on patient prioritisation required a different approach. Instead of exploring optimal scheduling policies through queuing theory and job scheduling [7, 28, 33], we concentrated on assigning patients to appropriate pathways. Patient prioritisation processes affect workload by shaping decisions about service speed and capacity allocation [26]. Notably, studies [20], have highlighted the dilemma in patient prioritisation: balancing patient-centred individual healthcare decisions with equitable resource allocation in healthcare management. Although clinical and non-clinical experts often have differing viewpoints, the literature extensively covers both aspects.

Priority setting, a key clinical stream relevant to this study, refers to the distribution of resources among competing programmes and patient groups. While priority setting occurs in every health system, research has mainly focused on the macro (national) and micro (bedside) levels, neglecting the meso (institutional, hospital) level despite hospitals' critical role in healthcare delivery and resource absorption [34]. Understanding how hospitals set priorities and the factors influencing resource allocation is imperative. Priority setting for older patients with chronic conditions or multimorbidity is complex, as disease-specific guidelines often do not apply [35]. Care-related goals for frail older adults vary and often include well-being alongside health and functioning [36], necessitating the integration of priority setting with complex care interventions. Priority setting at a systemic level influences clinical-level patient prioritisation.

A robust prioritisation system is essential for an efficient healthcare environment, focusing on (a) strategic patient placement and pathway assignment and (b) resource allocation. Operations management studies on patient placement, assignment, prioritisation and triaging, considering resource and capacity planning, are particularly relevant to this study. Patient prioritisation and triage have been applied in various contexts, including ED [35], acute and elective surgery [20, 23] and intensive care [37, 38]. Various studies have developed and extended multiple triaging systems and tools with the key objective of prioritising patients according to their acuity level [39], such as the Emergency Severity Index (ESI) [40], the Objective Primary Triage scale (OPTS) [31], the Adaptive Process Triage (ADAPT) [41] and the Patient Acuity Category Scale (PACS) [42]. Furthermore, tools using interRAI assessments have been developed to accurately determine a person's clinical and social support needs and prepare care plans. Few studies have developed older-persons-specific prioritisation tools [43] and existing tools often lack flexibility, emphasising the need for enhanced tools with comprehensive operational and health-related definitions [44]. This improvement would increase reliability and help identify factors associated with patient admission risks and successful outcomes.

Patient prioritisation significantly impacts patient flow through resource and capacity planning. Fast Track pathways, a form of server capacity prioritisation, have been extensively studied in ED management literature. While there is substantial literature on patient prioritisation in EDs [23], only a few studies have developed similar tools for older patients with a focus on resource management [32, 45]. Research studies have utilised data-driven methods such as simulation modelling [32], stochastic modelling [46], Markov decision process [47] and other optimisation models [48] to address these challenges. Although some studies have developed triaging and prioritisation tools for older patients[49, 50], the literature lacks a practical prioritisation tool for older patients that incorporates specialised care models like Fast Track alternatives and considers resource availability.

Evidence indicates that prioritising high-risk patients at discharge may be too late for effective interventions. However, early identification is increasingly feasible with detailed EHRs [29–31]. AI models can offer better prevention, diagnosis and treatment, with developments in cancer detection [51], disease management using robotics [52] and other patient safety factors [53]. AI technologies have assisted clinicians in decision-making [54, 55] with screening tools developed for early cognitive impairments [56, 57] and other older patient health problems, such as fall risks [58] and urinary tract infections among patients with dementia [59]. While some research has explored innovative AI/ML models for patients in general [60], the literature on intelligent patient prioritisation tools for older patients is limited [61, 62]. No previous studies have developed AI/ML models for older patient prioritisation to complex and non-complex pathways. This study not only creates a patient prioritisation tool for frail patients but also an interRAI-based solution for acute geriatric patients. Furthermore, no prior studies have developed a prioritisation tool that incorporates specialised care models like ACE as an alternative pathway. While applicable to New Zealand, this tool can be utilised for older patient prioritisation internationally. A summary of the literature review is presented in Table 1.

**Table 1:** Summary of the literature review

Reference	Domain	Model Type	Utilised Model	Patient Prioritisation	AI/ML Models	Older Patients	Specialised Alternative Pathway	Integration with EHR Systems
Ferrand et al. (2018)	ED/Ward	Operations Research	Mixed-Integer Programming	✓			✓	✓
Saghafian et al. (2015)	General	Stochastic Modelling	APOMDP	✓			✓	✓
Chakshu and Nithiarasu (2022)	Scheduling	Queueing Theory	Multi-Class Queuing Model					
Conner-Spady et al. (2004)	Scheduling	Job Scheduling	Dynamic Scheduling Algorithm					
Ding et al. (2019)	General	Operations Research	Linear Programming	✓				✓
Aseel et al. (2020)	General	Policy Analysis	Health Policy Framework	✓				
Déry et al. (2020)	General	Operations Research	Simulation-Based Optimization	✓			✓	
Li et al. (2023)	General	Policy Analysis	Cost-Effectiveness Analysis	✓				
Skirbekk et al. (2017)	General	Health Economics	Cost–Benefit Analysis	✓			✓	
McKneally et al. (1997)	General	Health Policy	Ethical Framework	✓				
Barasa et al. (2015)	General	Health Management	Resource Allocation Model	✓				
Martin et al. (2003)	General	Health Economics	Economic Evaluation	✓				
Shah (2009)	Geriatrics	Health Management	Priority Setting Framework	✓		✓		
Bethell et al. (2019)	Geriatrics	Health Management	Integrated Care Model	✓		✓		
Hunold et al. (2016)	Geriatrics	Health Policy	Policy Implementation Strategy	✓		✓	✓	
Vermunt et al. (2017)	Geriatrics	Health Management	Care Coordination Model	✓		✓	✓	
Robben et al. (2011)	Geriatrics	Health Policy	Chronic Care Model	✓		✓		
Azeez et al. (2013)	General	ADAPT	Adaptive Process Triage	✓			✓	
Hendin et al. (2018)	General	Predictive Analytics	Logistic Regression	✓				✓
LaMantia et al. (2013)	General	Data Mining	Decision Trees	✓				✓
Saghafian et al. (2014)	General	Stochastic Modelling	Discrete Event Simulation	✓				
Elalouf and Wachtel (2022)	General	Operations Research	Decision Support Systems	✓			✓	
Saghafian et al., 2015	General	Simulation Modelling	Monte Carlo Simulation	✓			✓	
Ahmed and Alkhamis (2009)	General	Stochastic Modelling	Queuing Network Model	✓				
Ekdahl et al. (2015)	General	Decision Support	Expert Systems	✓				
Zlotnik et al. (2016)	General	Markov Decision Process	Markov Chains	✓				
Marshall et al. (2001)	General	Optimisation Models	Integer Programming	✓				
Lu and Wedig (2013)	General	Optimisation Models	Genetic Algorithms	✓				
Abe et al. (2016)	General	Triaging Tools	Triage Scoring Systems	✓				
Gretarsdottir et al. (2021)	Geriatrics	Health Management	Care Pathway Analysis	✓		✓		
Abdel-Hafez et al. (2023)	General	AI/ML Models	XGBoost	✓	✓		✓	✓
Delshad et al. (2021)	General	AI/ML Models	Convolutional Neural Network (CNN)	✓	✓		✓	✓
Lee et al. (2021)	General	AI/ML Models	Recurrent Neural Network (RNN)	✓	✓		✓	✓
Marchiori et al. (2020)	General	AI/ML Models	Support Vector Machine (SVM)	✓	✓		✓	✓
Xie et al. (2021)	General	AI/ML Models	Random Forest	✓	✓		✓	✓
**This Study**	**Geriatric/Older Patients**	**AI/ML Models**	**Ensemble AutoML**	**✓**	**✓**	**✓**	**✓**	**✓**

Ultimately, the studies in Table 1 show that most existing work addresses only subsets of the problem: operations research and priority-setting models typically focus on resource allocation or patient flow without AI/ML or geriatric-specific design, while AI/ML studies often use EHR data but are not tailored to older patients or specialised alternative pathways such as ACE or Fast Track. Only few approaches integrate patient prioritisation, advanced ML, routine EHR data and explicit consideration of older-persons’ pathways within a single framework. Therefore, this study represents a novel combination of these dimensions, positioning REACH as one of the first ensemble AutoML models to support geriatric patient prioritisation into complex and non-complex pathways using EHR and interRAI data while accounting for specialised alternative pathways.

## Reach in action: Geriatric patient prioritisation

This study introduces Recognising Episodes of Acute Complexity in Health (REACH), a novel ML-based tool for prioritising older patients and assigning them to appropriate pathways and urgency categories. This section provides an overview of the ML pipeline and the details of the algorithms. The outcomes and findings from REACH for this case study are presented, followed by a discussion.

### Machine learning pipeline and model outcomes

This section presents an overview of the REACH pipeline, including ML preprocessing and the primary techniques used to create the predictive model. Advances in Artificial Intelligence (AI), particularly Machine Learning (ML), have enabled intelligent applications that provide predictive decision support [33, 63]. Many developers of AI systems now recognise that it can be far easier to train a system by showing it examples of desired input–output behaviour than to program it manually by anticipating the desired response for all possible inputs. A learning problem can be defined as the problem of improving some measure of performance when executing tasks, through some type of training experience [64].

**Fig. 1:**
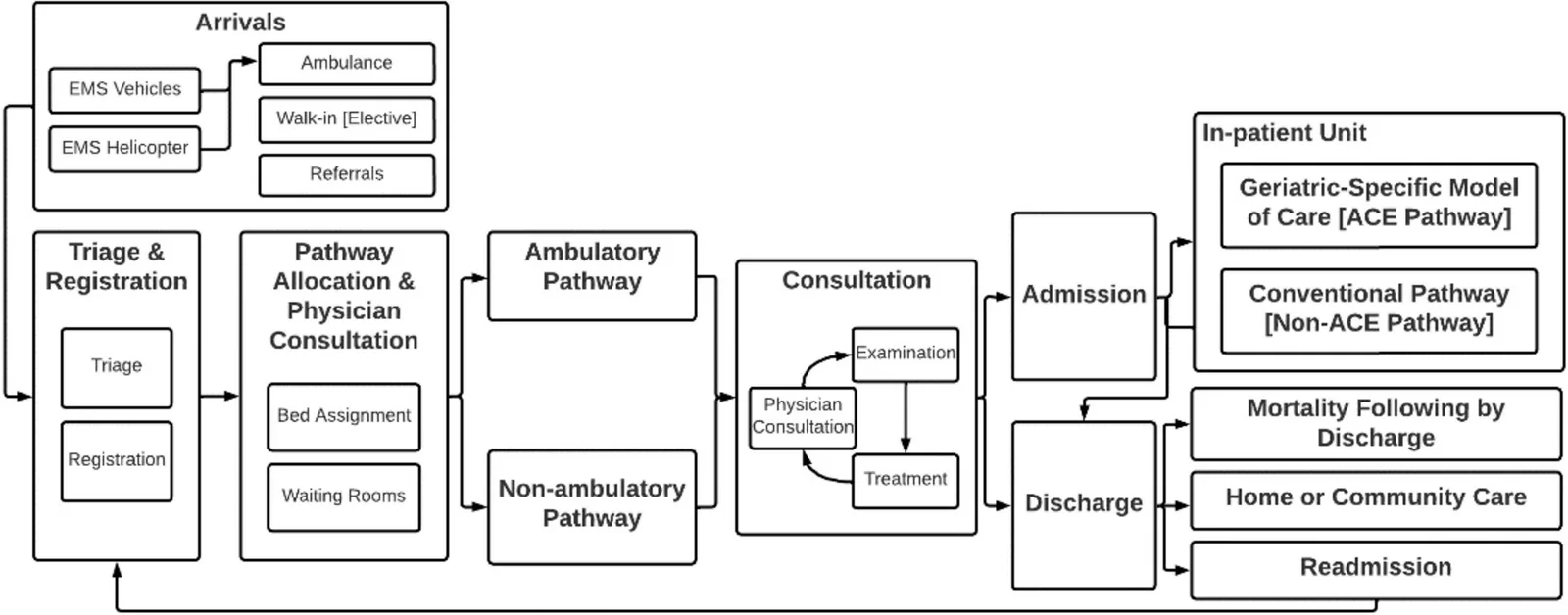
Patient flow from the emergency department to inpatient units under the reach framework

REACH approach categorises patients into two pathways, complex and non-complex, enabling tailored care delivery according to each patient’s needs, optimising outcomes and resource utilisation. REACH uses ML algorithms to evaluate patient data and assign them to the appropriate pathway based on the estimated probability of complexity. This probability guides the prioritisation process to ensure that patients most in need of immediate or intensive care are appropriately assigned. REACH accounts for resource allocation limitations during patient assignment and prioritisation, ensuring care distribution does not exceed available capacity. This balance between healthcare demand and finite resources such as hospital beds, staff and equipment is vital. The primary model for REACH is based on patient flow from the ED to inpatient wards. As shown in Fig. 1, older patients enter the system via the Emergency Department and are routed under the REACH framework into either a complex Older Persons and Rehabilitation (OPR) pathway or a non-complex inpatient pathway, with the Green/Yellow/Red REACH category determining the destination ward subject to ward capacity.

To address complex modelling tasks with ML models, especially in cloud and distributed environments, the model's structure is decomposed into interconnected blocks combined into a workflow. Although ML enables computers to learn without explicit programming, designing effective ML pipelines often requires considerable experience with algorithms, expert knowledge of the problem domain and brute-force search to achieve successful implementation and performance [64]. In response to this challenge, several AutoML methods have been developed over the years [65].

Although ML in healthcare is an active research topic, most health data collected is never used to build predictive models integrated into clinical settings; only 15 percent of hospitals routinely use ML even for limited purposes [66, 67]. A primary reason is that, despite ML's demonstrated benefits, successful utilisation requires extensive effort from human experts, since no algorithm performs well on all possible problems. Additionally, data and human expertise are often not readily available, especially in healthcare settings. Therefore, devising and deploying ML solutions is challenging, beginning with a lengthy data provisioning process, requiring collaboration between ML experts and domain experts and involving continuous back-and-forth[68]. Automating some components that require human expertise would allow the healthcare industry to build, validate and deploy ML solutions more readily, hence improving healthcare quality for patients. Motivated by this goal across industries, AutoML has emerged as a new research field to optimize parts of the ML pipeline automatically.

The adoption of AutoML in healthcare faces notable challenges, primarily due to unreliable and non-interoperable EHR data (originally designed for billing rather than clinical analytics) and the need for system transparency. These data issues complicate the use of AutoML, as models trained on such data may not perform optimally and the black-box nature of AutoML systems can lead to a lack of trust among healthcare professionals who require transparency in clinical decision-making.

AutoML automates the selection of the best ML models and tuning their hyperparameters to optimise performance for a given dataset. This is achieved through black-box optimisation, where the goal is to find the optimal configuration from a vast space of possible ML configurations without explicit knowledge of the underlying function being optimised. The optimisation problem at the core of AutoML can be described as a search for the optimal set of hyperparameters (${\lambda }^{*}$) that maximise a performance metric $F\left(\lambda \right)$ of the ML model and this problem can be presented as Eq. (1).

1 $${\lambda }^{*}=arg\underset{\lambda \in \Lambda }{\mathrm{max}}F\left(\lambda \right)$$

where Λ represents the hyperparameter space, encompassing all possible configurations that can be used to train a machine learning model. The space is typically high-dimensional and can include continuous, categorical, or integer hyperparameters. The performance metric $F\left(\lambda \right)$ is based on metrics such as accuracy, mean squared error, evaluated on a validation set or through cross-validation. It quantifies the effectiveness of a particular configuration (λ). Accordingly, solving the optimisation problem involves searching through the hyperparameter space Λ. This study used the grid search strategy to develop REACH model. Grid search is a systematic exploration of the hyperparameter space by evaluating a grid of hyperparameter combinations. Consider an ML model with two hyperparameters, ${\lambda}_{1}$ and ${\lambda}_{2}$, where ${\lambda}_{1}\in A=\{a,b,c\}$ and ${\lambda}_{2}$ $\in B=\{x,y\}$. The grid of hyperparameter combinations, denoted as Λ is defined as $\Lambda =A\times B=\left\{\left(a,x\right),\left(a,y\right),\left(b,x\right),\left(b,y\right),\left(c,x\right),\left(c,y\right)\right\}$. Then, for each combination $\lambda =\left({\lambda}_{1},{\lambda}_{2}\right)$ in Λ, the model performance $F\left(\lambda \right)$ is evaluated.

Selecting the suitable ML algorithm and tuning its hyperparameters is crucial for creating practical solutions, as no single algorithm excels across all problems and datasets. The Combined Algorithm Selection and Hyperparameter-tuning (CASH) problem addresses these challenges by automating the identification of the most effective ML models for specific tasks [69]. The CASH problem seeks to automate two critical decisions in the ML pipeline: selecting the most appropriate ML algorithm from a portfolio of candidates A and tuning its hyperparameters Λ to optimise performance. This dual objective addresses a significant challenge, where both choices can dramatically impact the effectiveness of the final model. Considering a set of available algorithms A and the hyperparameter space corresponding to the j th algorithm, there can be a loss function $L\left(F(x)\right)$ that is used to evaluate the performance of each algorithm ${A}^{j}$ with hyperparameters λ on the training ${D}_{train}^{i}$ and test ${D}_{test}^{i}$ datasets. The goal is to minimise loss, indicating better model performance, as demonstrated in Eq. (2).

2 $${A}^{*},{\lambda }^{*}=arg\underset{{A}^{j}\in A,\lambda \in \Lambda }{\mathrm{min}}\frac{1}{k}{\sum}_{i=1}^{k}L\left({A}_{\lambda }^{j},{D}_{train}^{i},{D}_{test}^{i}\right)$$

**Fig. 2:**
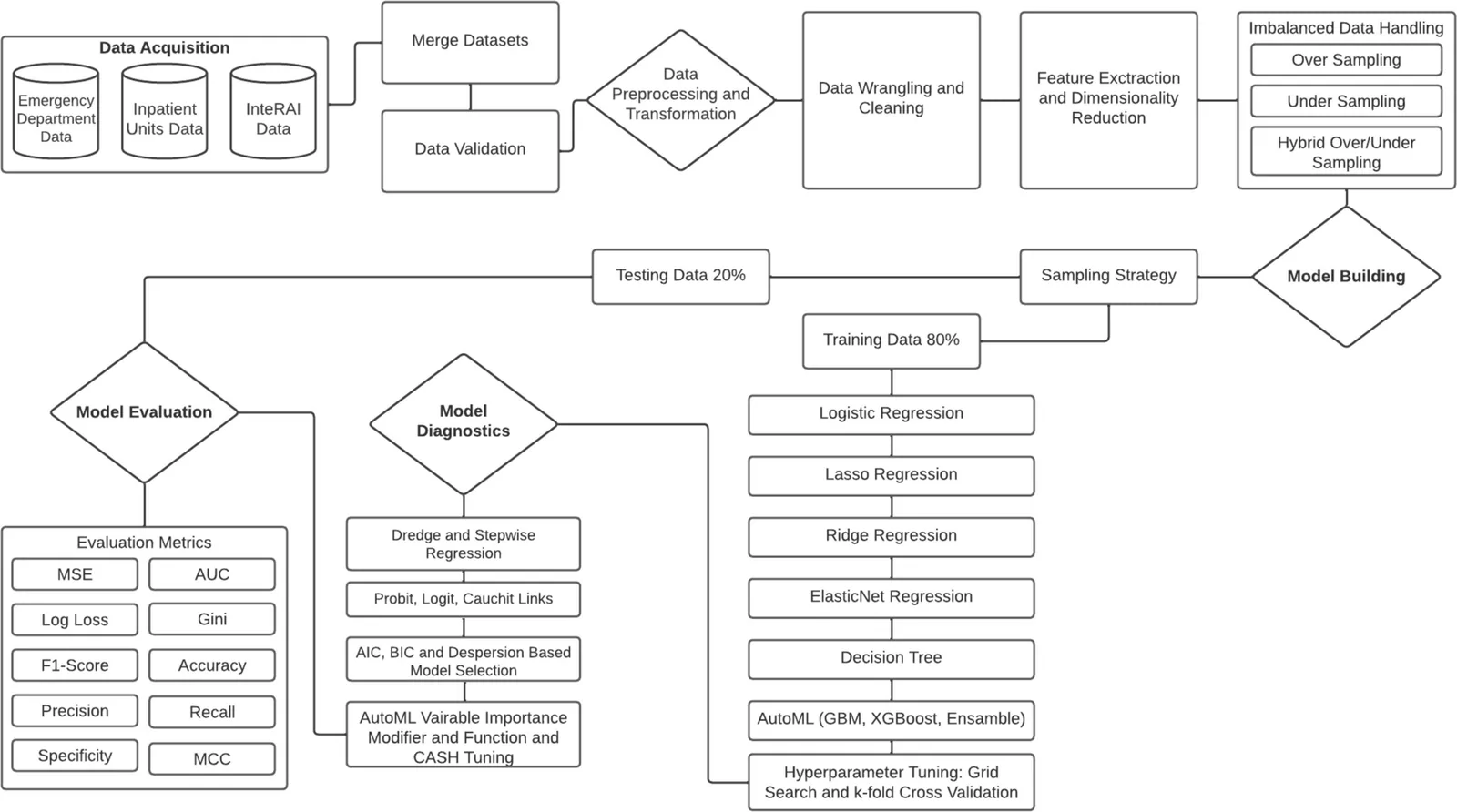
Reach machine-learning pipeline

Solving the CASH problem involves navigating a more extensive search space than hyperparameter tuning alone, requiring efficient exploration of both the algorithm space and the associated hyperparameter spaces, which can significantly increase computational burden. Ultimately, REACH is an AutoML model using a CASH optimisation model, consisting of an ensemble of ML models. The end-to-end composite architecture of the REACH machine-learning pipeline, including data ingestion, preprocessing, AutoML/CASH-based model selection, and the Green/Yellow/Red threshold output, is illustrated in Fig. 2.

To summarise, in practical terms, REACH operates in two layers. First, in this study, a retrospective planning layer uses de-identified historical EHR and interRAI data to train the AutoML model and estimate a complexity probability for each completed admission; these probabilities are then used in the simulation to design threshold policies and explore capacity scenarios for complex geriatric beds. Second, in a future real-time layer, the same trained model could be embedded in the hospital EHR so that, at or shortly after ED presentation, routinely available variables (e.g. age, ethnicity, triage code, presenting diagnosis, prior utilisation, interRAI history) are passed automatically to REACH, which returns a single complexity probability and an associated Green/Yellow/Red flag to be displayed on ED or bed-management dashboards as decision support for clinicians.

### Context definition: data sources and validation

This study utilises retrospective patient data from the EHRs of HNZ Waikato District, with ethical approvals obtained from relevant institutions. It adheres to the guidelines of the University of Auckland and the University of Waikato Human Research Ethics Committee. The dataset spans seven years (January 2016 to May 2023) and includes over 620,000 patients, merging records from ED, hospital admissions, interRAI and AR-DRGs. A de-identification process was employed to maintain patient confidentiality. While focused on older patients, the model is not limited to individuals aged 65 and above, thus including complex cases that might otherwise be excluded. Exclusions include paediatric outpatient care, incomplete medical records, hospitalisations outside the study period and deceased individuals to ensure data integrity.

The dataset features de-identified patient demographics (gender, ethnicity, age, domicile) which is important for prioritising care. It includes comprehensive EHR data from ED and inpatient units: admissions, discharges, urgency levels, mortality rates, patient transfers and length-of-stay. Diagnosis types are classified according to ICD-10, with complexity levels determined by AR-DRG and Patient Clinical Complexity Level (PCCL) indicating clinical severity. The Australasian Triage Scale (ATS) specifies maximum ED wait times and readmission data covers occurrences within 28 days to six months post-discharge. Hospital costs, including primary output measures and capital indicators like bed numbers, help determine costs per length-of-stay and clinical episode. Capacity and resource management details, such as occupied beds and available resources, are included.

The interRAI tool was employed to ascertain a range of patient characteristics, from clinical aspects to social support, thereby facilitating the formulation of comprehensive care plans. This assessment is highly valuable for healthcare professionals, encompassing various sub-assessment types. Furthermore, the calculation of readmissions was based on the respective discharge and admission dates for each patient episode. This encompassed events from admission to discharge, including transitions between different wards. For the simulation stage and capacity planning part of the algorithm, resources from the Older People and Rehabilitation (OPR) ward were included, where resources are logically segregated and allocated across prioritised patient streams within a department. Data validation was performed before preprocessing to ensure data integrity for subsequent stages, identifying and rectifying inconsistencies or errors.

### Data preprocessing: feature extraction, dimensionality reduction and data imbalance handling

**Fig. 3:**
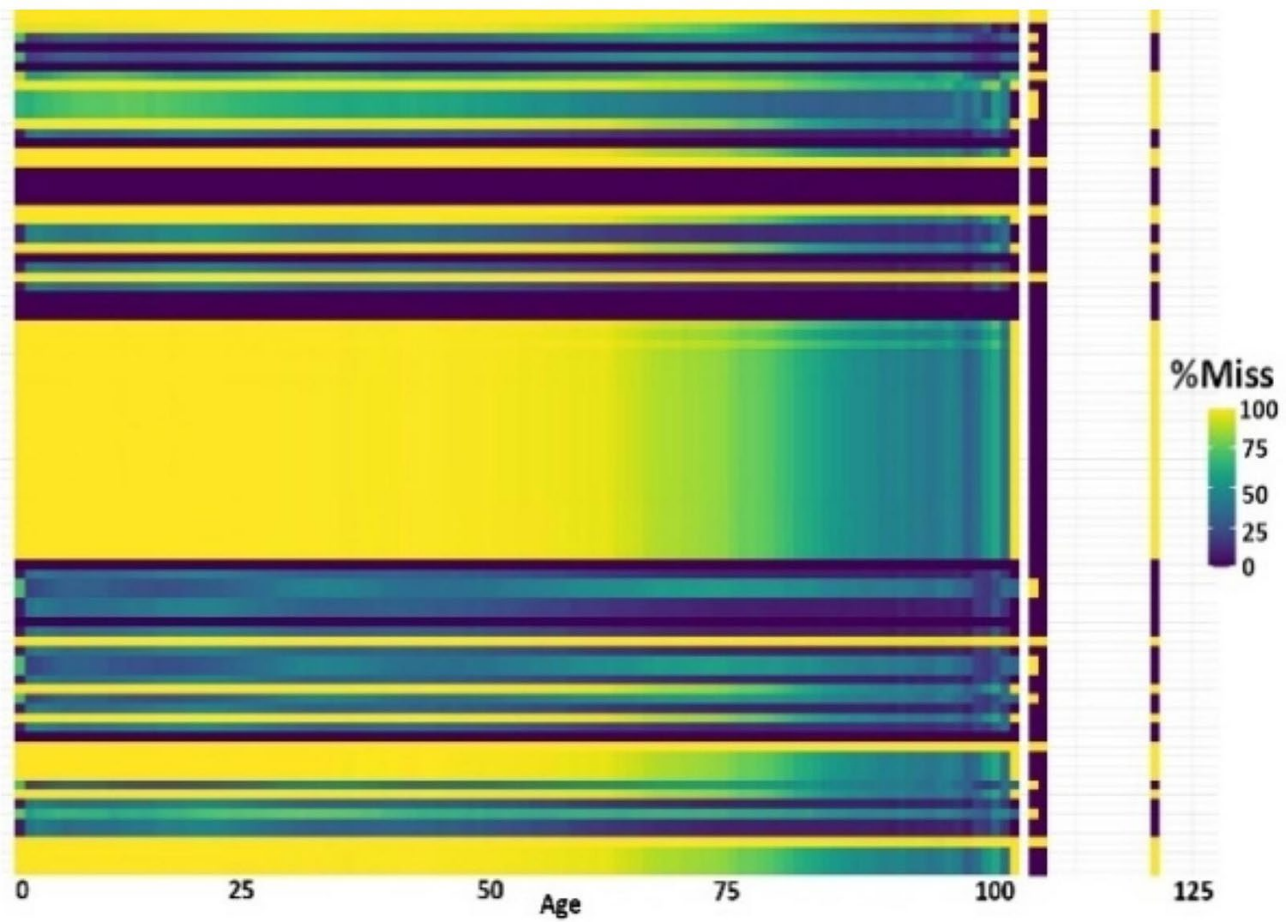
Missingness map of the initial dataset

Data preprocessing addressed challenges such as variable overload, data imbalance, missing values, outliers and noise, which can limit model performance. Preprocessing improves data quality and aligns it with algorithmic requirements, thereby enhancing accuracy and efficiency in predictions. This structured approach, from data validation to model development, is vital for establishing a robust pipeline. The training process utilised historical data from 867,764 patient episodes (January 2016 to May 2023). The focus was on admitted patients, excluding those discharged directly from the ED to prioritise ward allocations. Although age is a critical variable, it may not always be suitable for distinguishing patients due to ethnic differences in mortality rates between Māori, Pasifika and New Zealand European populations. As a result, patients under 25 years of age were excluded, which improved the dataset's quality for training purposes. Figure 3 illustrates the percentage of missing data across age groups in the initial dataset. The darker bands represent areas with a higher proportion of missing values, which were particularly prevalent among older patients and in specific wards. Notably, the dataset lacks records for patients aged 65 and above in the OPR ward before 2018, as this ward was established that year. This visualisation highlights substantial gaps in data completeness that could undermine model reliability if left unaddressed.

**Fig. 4:**
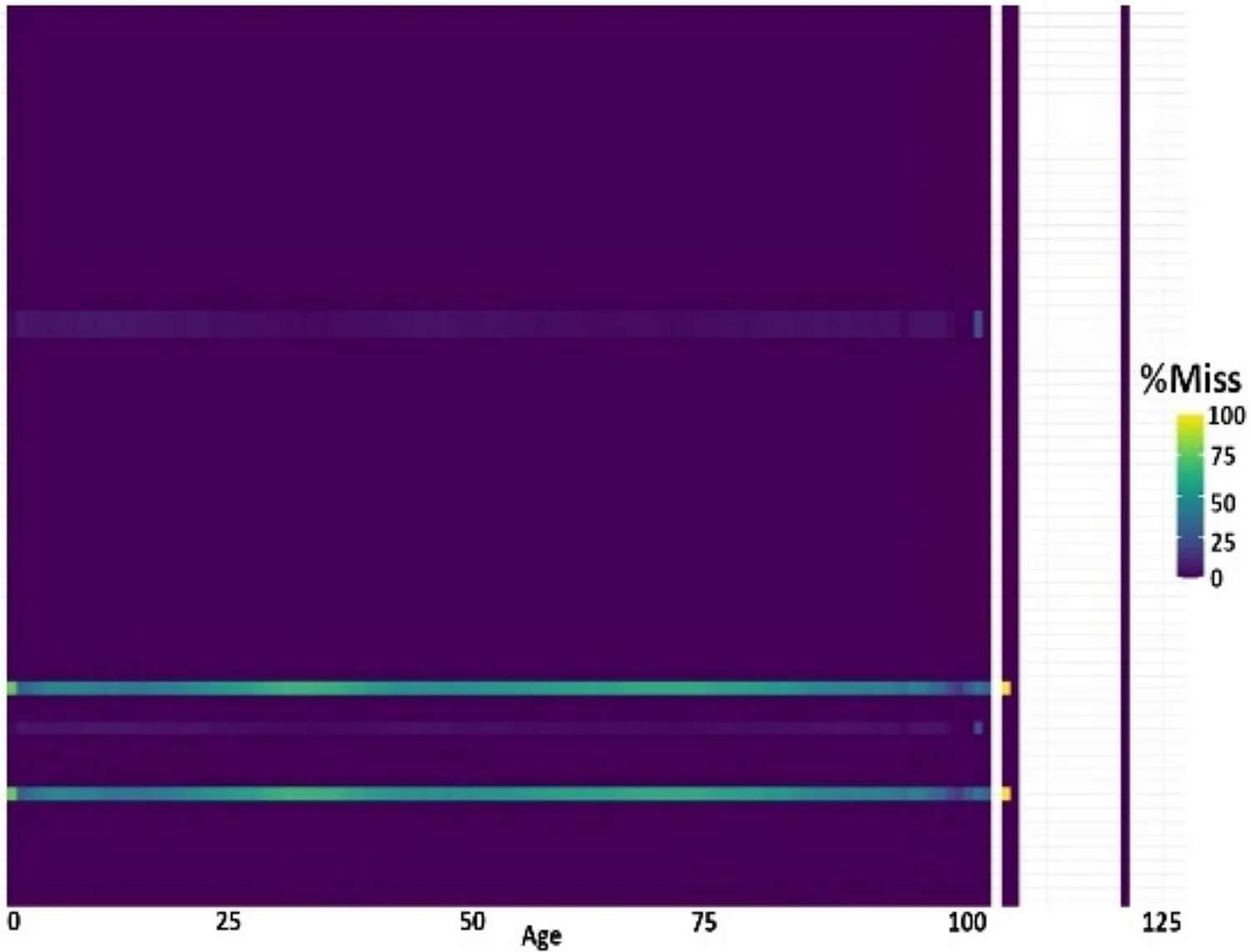
Missingness map of the dataset after preprocessing

A missingness map was used to identify and remove redundant data, as shown in Fig. 3, to improve the dataset's robustness. Preprocessing was carried out using R programming, specifically employing the Amelia, preProcess and ISLR packages. The absence of data for older patients in the OPR ward before 2018 reflects the timeline of the ward's establishment. Figure 4 depicts the extent to which missing data was reduced following preprocessing. Initially, as seen in Fig. 3, 64% of the dataset contained missing values. After applying data cleaning methods, this was reduced to only 2%. The lighter bands in Fig. 4 indicate areas with little to no missing data, demonstrating the success of these preprocessing techniques. To further address missing data, entities with a high proportion of N/A attributes were removed. Additionally, episodes and patients with fewer than one ward transfer and lacking supplementary information (e.g., ED admissions or day-stays) were excluded.

Furthermore, data missingness was evaluated to determine whether it was Missing Completely at Random (MCAR), Missing at Random (MAR), or Not Missing at Random (NMAR). Analysis using Little’s MCAR test indicated that the missing data were not MCAR; hence, further investigations were conducted to understand the patterns and mechanisms of missingness. Our assessment revealed that a significant portion of the data was MAR, influenced by observable variables such as patient age (under 25) and department (OPR not being established before 2018 and the patient discharged from ED and not admitted to an inpatient unit). For cases identified as NMAR, where data missingness could bias the model by excluding more complex cases, we have not excluded any events. Consequently, from the initial pool of 867,764 entities, the cleaned dataset now encompasses 653,942 episodes with minimal missing details, maintaining model integrity.

By forming a wrangled and cleaned dataset, the algorithm had a reliable foundation to build upon. Furthermore, from the 653,942 episodes within the cleaned dataset, only 2,231 were within the OPR ward, with the rest spread across other wards, presenting significant data imbalance that necessitated appropriate handling for improved prediction accuracy. This study addresses this imbalance by employing over-sampling, under-sampling and a hybrid approach, effectively rectifying the data distribution using H2O, autoresampling, mlbench and caret packages. The last stage of preprocessing involved feature extraction and dimensionality reduction to identify the core attributes of the model. Recursive Feature Elimination (RFE) with a Least Squares Support Vector Machine (LS-SVM) classifier was implemented through random stratified sampling [70]. For unbiased performance evaluation, the sample was divided into training (80%) and testing (20%) sets across five bootstrapped segments, maintaining a consistent prevalence ratio. The termination criteria for the RFE-LS-SVM were based on predefined accuracy thresholds and Cohen’s kappa. For the feature extraction and dimensionality reduction stage of the study sigFeature and caret packages were utilised. The model’s accuracy was consistently high, ranging between 82.77% and 93.74%, with a Kappa value of 0.43 to 0.62, affirming the stability of the RFE algorithm. Ultimately, the algorithm removed 39 attributes from 66, identifying core criteria for prioritisation. The final selection was validated using the significance importance of the attributes extracted from the ML model. REACH was created based on the attributes extracted from this final data preprocessing phase, as shown in Table 2.

**Table 2:** Summary of the core criteria within reach

Criteria	Description
Age	The age of the patient at the time of hospitalisation. This criterion is an essential demographic factor influencing various aspects of patient care and treatment decisions
Length of Stay	The duration of a single episode/event of hospitalisation. Inpatient days are calculated by subtracting day/time of admission from day/time of discharge
Admission Type	Hospital-defined code representing the circumstances under which the patient was or will be admitted, including acute, arranged, waiting list and external
DRG Resource Utilisation Code	The last digit is a split indicator that ranks Australia Refined Diagnosis Related Groups (AR-DRG) based on their consumption of resources. Higher the code number indicates lower consumption
Triage Code	A triage tool which is the first respond that the medical personnel use during an incident. A higher code number indicates lower consumption
Number of Stops	Total number of transfers and stops within different wards for a single episode/event of hospitalisation
Total Cost	Overall cost incurred during a single episode/event of hospitalisation. It includes expenses related to medical procedures, medication, room charges, diagnostic tests and other hospital services provided to the patient
Number of Comorbidities	The count of additional medical conditions or diseases that a patient has in addition to the primary condition for which they are being treated. Comorbidities can impact the complexity of care and resource utilisation
interRAI Assessment Type	The assessment type gives some insights into the patient’s complexity levels and assessment. This attribute helps with identifying the most complex patients
Patient Clinical Complexity Level	The patient clinical complexity level comes out of the DRG grouper program and the clinical severity is identified within the record
Emergency Department Revisit	The rate at which patients return to the emergency department for medical care after a recent hospital/ED discharge. It helps assess the effectiveness of post-discharge care and management
Pain Scale	Scale that attempts to define levels of pain. The scale is highly predictive of pain on the Visual Analogue Scale. Pain that is adequately managed is not featured on the scale
Depression Rating Scale	The Depression Rating Scale (DRS) is used as a clinical screen for depression. The higher the score the stronger the clinical indicator
Communication Scale	This scale summarises the person’s ability to communicate with others and to comprehend information
Pressure Ulcer Risk	This scale identifies persons at various levels of risk for developing a pressure ulcer to target risk factors for prevention
Maple Scale	MAPLe differentiates people into five priority levels, based on their risk of adverse outcomes. The lowest priority levels are considered self-reliant and have no major functional, cognitive, behavioural, or environmental problems
CHESS Scale	The Changes in Health, End-Stage Disease, Signs and Symptoms Scale was designed to identify individuals at risk of serious decline. It can serve as an outcome where the objective is to minimise problems related to decline in function, or as a pointer to identify persons with unstable conditions
Aggressive Behaviour Scale	This scale provides a measure of aggressive behaviour. Higher scores indicate greater frequency and diversity of aggressive behaviour
Cognitive Performance Scale	The Cognitive Performance Scale (CPS) combines information on memory impairment, level of consciousness and executive function
Activity of Daily Living (Short Form)	Scale that provides a summary of the person’s ability to perform ADLs. It is based on 7 ADL items – personal hygiene, toilet use, locomotion, eating, dressing and transferring and bed mobility. The higher the score, the greater the difficulty of performing activities
Activity of Daily Living (Hierarchy Scale)	This Scale groups activities of daily living according to the stage of the disablement process in which they occur. Early loss ADLs (for example, dressing) are assigned lower scores than late loss ADLs (for example, eating)
Instrumental Activity of Daily Living Performance	This Scale is based on a sum of eight items: meal preparation, ordinary housework, managing finances, medications, phone use, stairs, shopping and transportation. Higher scores indicating greater dependence
Instrumental Activity of Daily Living Capacity	
28-days Readmission	The rate at which patients are readmitted to the hospital within a specific period, i.e., 28 days, three, six-months after their initial discharge. It serves as an indicator of the effectiveness of the initial treatment and discharge planning
3-Months Readmission	
6-Months Readmission	

### Model development, evaluation and hyperparameter tuning

There are many complementary blocks involved in the pipeline (Fig. 2). For example, different feature selection algorithms are applied to the same dataset to extract more valuable features from the data. However, building composite pipelines (optimal selection and tuning of the blocks and connection between them) is a complicated and time-consuming task even for experts in ML. That is one of the main reasons that REACH was built upon the fundamentals of CASH. This approach facilitates the selection of the best-performing model based on the data and problem specifics. To further enhance the robustness of REACH, extensive testing and validation phases are incorporated, ensuring that each selected feature contributes positively to the overall predictive power of the model. This rigorous validation process not only fine-tunes the model for higher accuracy but also ensures its generalisability across different conditions.

The primary response variable of the model is whether a patient is admitted to a complex OPR ward, with the explanatory variables detailed in Table 2. Several of these explanatory variables, such as length-of-stay, readmission and total cost, are only fully observed by the end of an admission rather than at the point of ED triage. In this study, REACH was therefore developed and evaluated as a retrospective decision-support tool using historical inpatient records, with these variables used to learn and characterise the construct of complexity and its operational and clinical correlates over the whole episode of care. In other words, the current version of REACH is designed to support offline policy design, threshold setting and capacity planning for complex geriatric pathways, rather than to be deployed directly at the bedside during triage. A future real-time implementation would require retraining the model using a restricted feature set that includes only information available at or before ED presentation (for example, demographic characteristics, current triage code, presenting diagnosis and prior utilisation and interRAI history).

For each patient in the group, REACH determines their pathway assignment by calculating the complexity probability. This probability not only determines the initial pathway assignment but also prioritises patients within each complexity category. Green indicates a non-complex patient; yellow suggests a borderline case requiring human decision-making; red signifies a high complexity threshold, meaning the patient should be assigned to a complex ward. REACH serves as a decision support tool, assisting clinicians in allocating patients to complex or non-complex care pathways. Therefore, it is possible to adjust the threshold to finer scales (like a 6-point or 8-point scale) using the complexity probabilities. This ensures that patients with higher complexities are given precedence, provided that the necessary resources are available.

Hyperparameter tuning is then conducted to fine-tune the model parameters for optimal performance. This involves an exhaustive search over a predefined range of hyperparameter values to identify the most effective settings for the algorithms in use. Hyperparameter tuning was conducted to maximise the K-fold cross-validation Area Under the Curve of Receiver Operating Characteristic (AUC-ROC), a critical measure for assessing the performance of classification models. The model’s overfitting tendency post-cross-validation was a key refinement indicator. The best algorithm and its hyperparameters were selected through the AutoML process. This process involves running an optimisation loop over a set of algorithms and their hyperparameter spaces to find the combination that minimises the average loss across k-fold cross-validation on training and testing datasets. This process directly impacts how the probability of a patient being assigned to a complex pathway is calculated, ensuring the decision is based on the most effective model identified by the AutoML process. This approach allows the model to dynamically adapt to the best ML strategy for the classification phase, enhancing the model’s accuracy and reliability in assigning phases. All computational experiments and calculations were performed in R programming language using a suite of packages, including H2O AutoML, xgboost, ISLR, rpart, autocart and caret.

A descriptive analysis of the pre-processed data indicates that the hospital received referrals for 261,773 patients, encompassing 653,942 patient episodes and events. Of these, there were 138,913 females (53.1 percent) and 122,837 males (46.9 percent), with an average age of 40.41 years. The ethnicity breakdown showed a majority of NZ European patients at 137,280 (52.4 percent), followed by Māori at 66,798 (25.5 percent), with other ethnicities including Indians, Chinese and Africans making up the remainder, collectively accounting for 22.2 percent. The descriptive analysis showed an average length-of-stay of 2.20 days, where admissions were primarily acute (57 percent), with the rest being waiting list admissions, arranged admissions and other. Discharges were mostly from inpatient units (66.1 percent) and the emergency department (19.7 percent), including 2,529 (1 percent) deceased.

Of the total, 10,447 patients were accommodated in short-stay wards, with 1,065 in the Acute Medical Unit (AMU) and 9,382 in Respiratory, Medical, Gastro and Neurology wards. Additionally, 2,170 patients were housed in OPR wards, with 637 in the complex OPR ward and 1,533 in Stroke, Orthopaedic rehabilitation and Assessment, treatment and rehabilitation OPR. The complex OPR ward housed a majority of female patients (53.7 percent) and NZ Europeans (67.2 percent), with 25.5 percent Māori and Pasifika patients. Patients’ average age was 77, higher than other wards. Resource usage stood at 46.6 percent, based on AR-DRGs and 67 percent of patients were discharged post-recovery. The ward registered a low case fatality rate of 6.3 percent and high PCCL of between three and four, consistent with ACE and GEMU principles. Despite a high comorbidity prevalence, with 35 percent having at least one severe PCCL condition, 98.7 percent of patients underwent extensive evaluation with a minimum of four interRAI instrument assessments. Utilising the REACH pipeline, 62 different models were trained, including ensemble variations of the models within the AutoML process. Table 3 summarises the top five performing models on the held-out test data, reporting AUC-ROC, log-loss, F1-score, accuracy, precision, recall, specificity, MCC and overall classification error. Grid search and five-fold cross-validation were also enabled within the algorithm. The H2O AutoML package was used as the primary interface for processing the data.

**Table 3:** Top five performing models extracted from the automl pipeline

Model	AUC	Log Loss	Gini	F1	Accuracy	Precision	Recall	Specificity	MCC	Overall Classification Error
Stacked Ensemble A	0.814	0.442	0.628	0.886	0.962	0.943	0.393	0.962	0.779	0.014
Stacked Ensemble B	0.807	0.440	0.615	0.879	0.956	0.921	0.342	0.956	0.723	0.023
Stacked Ensemble C	0.793	0.516	0.666	0.862	0.907	0.905	0.267	0.931	0.714	0.019
XGBoost A	0.784	0.496	0.611	0.851	0.895	0.902	0.241	0.925	0.711	0.027
XGBoost B	0.778	0.406	0.557	0.856	0.879	0.896	0.223	0.916	0.706	0.029

**Stacked Ensemble A**; Hybrid Balanced Data, Xgboost, GBM, ElascticNet, Ridge, fivefold CV, Hyperparameter Tuning using Grid Search. **Stacked Ensemble B**; Undersampled Balanced Data, Xgboost, GBM, ElascticNet, Ridge, fivefold CV, Hyperparameter Tuning using Grid Search. **Stacked Ensemble C**; Hybrid Balanced Data, Xgboost, ElascticNet, fivefold CV, Hyperparameter Tuning using Grid Search. **XGBoost A**; Hybrid Balanced Data, Xgboost, fivefold CV, Hyperparameter Tuning using Grid Search. **XGBoost B**; Oversampled Balanced Data, Xgboost, fivefold CV, Hyperparameter Tuning using Grid Search

Model performance is critically evaluated through various metrics. These metrics can be briefly defined as follows. **Area Under the Receiver Operating Characteristic Curve (AUC-ROC)**: This represents the model’s ability to discriminate between positive and negative classes. A higher AUC indicates better model performance. **Log Loss**: Quantifies the accuracy of a classifier by penalising false classifications, focusing on the probability estimates of the predictions. **Gini Coefficient**: A measure derived from the AUC, indicating the model discriminatory power. It assesses the inequality among the model prediction values. **F1 Score**: The harmonic mean of precision and recall, balancing the model precision and recall. **Accuracy**: The ratio of correctly predicted observations to the total observations, measuring the overall correctness of the model. **Precision**: The ratio of correctly predicted positive observations to the total predicted positives, assessing the model exactness. **Recall**: The ratio of correctly predicted positive observations to all actual positives, measuring the model completeness. **Specificity**: The proportion of true negative predictions in all actual negatives, indicating the model’s ability to correctly identify negatives. **Matthews Correlation Coefficient (MCC)**: A quality measure of binary classifications, capturing the correlation between observed and predicted classifications. **Overall Classification Error**: Reflects the proportion of all incorrect predictions over the total number of cases, providing an indication of the model misclassification rate. This evaluation ensures that the model reliably predicts patient categories and effectively prioritises them based on the estimated complexity. The stability and reliability of the model are confirmed through extensive testing, ensuring its efficacy in real-world applications.

The analysis of the AutoML pipeline output shows that Stacked Ensemble A emerges as the superior model among the top five performing models from 62 candidate models, as demonstrated by its performance across multiple classification metrics in Table 3. This model achieved the highest AUC-ROC of 0.814, indicating good discrimination between complex and non-complex cases, and the lowest log loss (0.442), reflecting well-calibrated predicted probabilities. The F1-score of 0.886, together with an accuracy of 0.962 and precision of 0.943, shows that the model maintains a favourable balance between correctly identifying complex patients and avoiding unnecessary complex admissions. Although recall is more modest (0.393), the high specificity (0.962) and MCC of 0.779 indicate a reliable classifier with a strong positive association between predicted and observed classifications. In the AutoML process, model selection was based primarily on AUC-ROC and log loss, with these additional metrics reported to provide a comprehensive view of classification performance.

Although Stacked Ensemble A achieved a low overall classification error (0.014), this figure is more meaningful when expressed in terms of patients and the types of mistakes the model makes. An error rate of 0.014 corresponds to roughly one misclassified patient in every seventy. After optimisation of the REACH thresholds, 131 of these cases were complex older patients who were not identified as such (false negatives), while 3,442 were patients flagged as complex who in practice did not require a complex geriatric bed (false positives). In other words, the overall error rate is low, but, because the non-complex group is much larger, it is dominated by misclassifications that mainly affect bed utilisation rather than safety–critical omissions. The other performance metrics can be interpreted in a similar way. The AUC of 0.814 indicates that in more than eight out of ten randomly chosen patient pairs (one complex, one non-complex), REACH assigns a higher complexity probability to the genuinely complex case. The high specificity (0.962) and precision (0.943) show that when REACH labels a patient as complex, this is usually correct, while the more modest recall (0.393) reflects the conservative design of the current thresholds, which prioritise avoiding unnecessary complex admissions over capturing every potentially complex case. In the simulation analysis, we partially rebalance this trade-off by tuning the thresholds to reduce missed complex patients (from 183 to 131 per year) and lowering false positives (from 5,534 to 3,442) while remaining within realistic ward capacity constraints. Together, these interpretations link the statistical metrics directly to patient prioritisation, bed allocation and the risk of delayed or inappropriate care for frail older adults.

The next stage is the simulation modelling phase with a primary goal to maximise the efficient use of complex care beds while ensuring patients are allocated appropriately based on their complexity. This can be done by establishing policy thresholds for REACH (Green, Yellow, Red), reflecting different levels of complexity for patients. The simulation’s objective is to support policymaking by providing a framework that can predict and manage patient flow based on their complexity, thus aiding in operational decisions that affect patient care and resource allocation.

### Findings and results: simulation analysis

In REACH, each patient's pathway is determined by assessing their complexity probability, calculated from individual patient characteristics and historical data. Patients are assigned and prioritised for the specialised ward based on their complexity probability. The model ensures that those with higher complexities receive attention first, provided that the necessary resources are available. The prioritisation mechanism is governed by two primary objectives: (a) categorising patients into complex and non-complex pathways and (b) prioritising patients in each pathway based on complexity probabilities. After selecting the best ML pipeline, thresholds are needed in REACH to define pathways based on patients' complexity probabilities. The primary goal of this phase is to maximise the efficient use of complex care beds while ensuring patients are allocated appropriately based on their complexity. Thresholds were chosen based on the resulting occupancy levels in the wards during the simulation. By simulating different scenarios and adjusting thresholds incrementally, optimal values were determined to ensure efficient resource utilisation and minimise patient delays.

This is achieved by establishing policy thresholds for REACH, reflecting different levels of patient complexity: Green indicates the patient should not go to the complex ward; Yellow suggests the patient is borderline and should be considered based on current complex ward availability and clinical advice; Red indicates the patient should go to the complex ward. Moreover, the policy thresholds can be improved based on simulation modelling outcomes, reflecting different levels of system capacity and demand. The thresholds for different strata are bespoke to the current capacity of the complex ward.

Suppose there are X patients within our historical data (which includes the date of admission, admitted wards and discharge date) over a period of a specific number of days (i.e., 365 days in this study). To focus specifically on the OPR ward, initial data processing was undertaken to exclude non-OPR ward data. This refined dataset tracked the date each patient was admitted to and subsequently discharged from, the OPR, with the discharge date determined by adding the length-of-stay in OPR to the admission date. To simulate and understand occupancy dynamics in the OPR ward, the patient allocation model was adjusted under varying capacities, considering the ward's specific structure. An occupancy vector, representing the length of stay or the number of days, can be initialised with a starting value of zero (indicating no initial occupancy at the beginning of the study period). This setup enabled dynamic calculation of occupancy based on patient flow and operational changes.

The simulation model treats the REACH complexity probability as its sole input from the predictive model. For each historical admission *i* in a 12-month data-trace, we take the patient-specific probability ${p}_{i}$ output by the stacked ensemble and apply a candidate pair of thresholds $\left({\eta}_{green},{\eta}_{red}\right)$ to assign that admission to Green, Yellow or Red. Admissions with ${p}_{i}$​ above the Red threshold are treated as “complex” and assumed to require a bed in the complex OPR ward; Yellow cases are admitted to the complex ward only if capacity is available; Green cases are always managed in non-complex wards. Arrival times and lengths of stay in the simulation are not generated from stochastic distributions, but taken directly from the observed admission and discharge dates and OPR length-of-stay for each episode. For each candidate threshold pair and assumed complex-bed capacity, the data-trace is replayed day-by-day to compute simulated ward occupancy and the numbers of correctly and incorrectly classified complex patients under that policy.

**Fig. 5:**
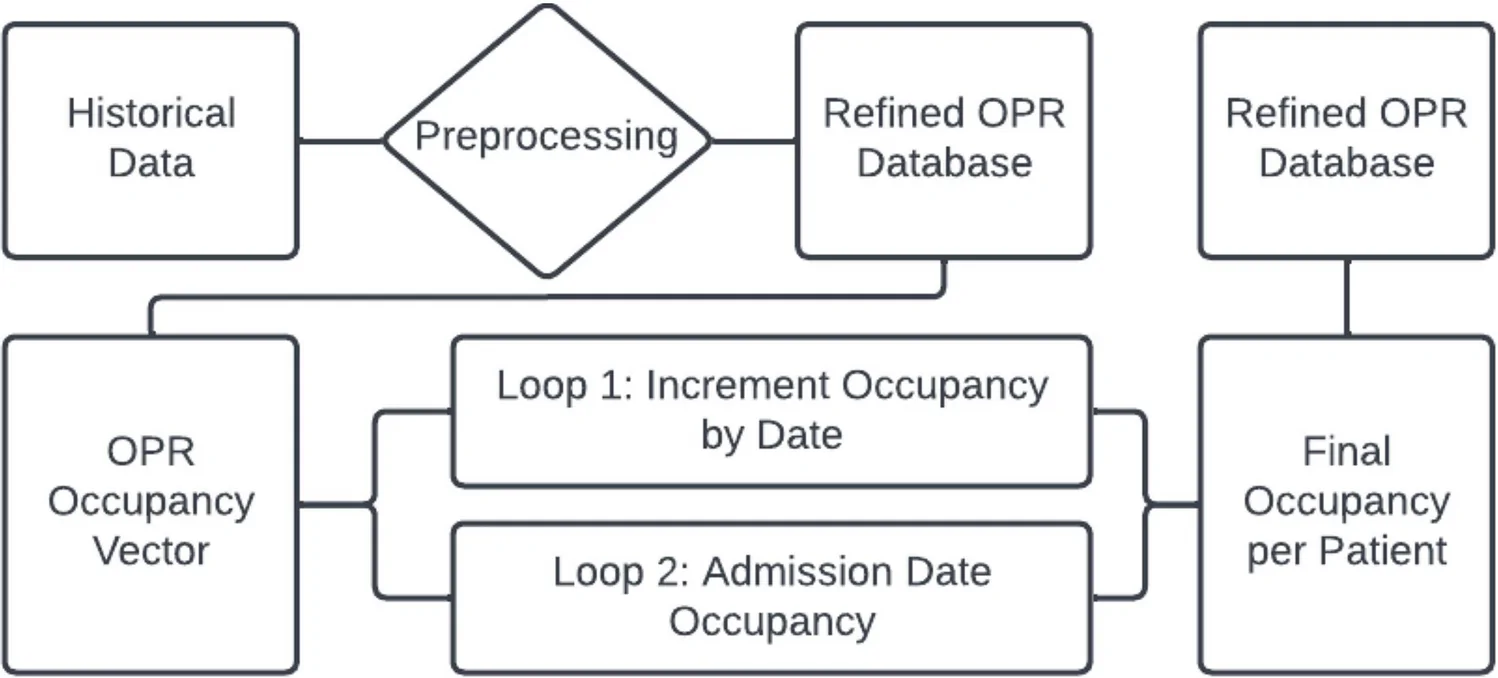
Conceptual diagram of the reach data-trace simulation

During the simulation, two primary loops processed the data. The first loop incremented the occupancy count for each day a patient was in the OPR ward. Patients who met the defined complex threshold were identified and assigned to the complex pathway and for each such patient, the occupancy from their day of admission j through j+l (where l is the length-of-stay) was increased by one. This method not only tracked the physical presence of patients but also highlighted the resource implications of managing complex cases. In the second loop, the occupancy count on each patient’s admission date was recorded to reflect the ward’s capacity at any given time. If a patient was admitted on a day that was already counted, the occupancy count was adjusted to reflect the new addition. For instance, if the ward count was 10 on day 15 and a patient was admitted on that day, the count would increase to 11. Figure 5 provides a conceptual illustration of the two-loop simulation process used to compute daily occupancy and misclassification counts for each candidate threshold pair.

This data-trace design relies on several explicit assumptions: (i) the empirical pattern of arrivals and lengths of stay over the chosen 12-month window is representative of typical demand; (ii) implementing REACH does not, in itself, change overall admission rates or case-mix; and (iii) complex-ward capacity can be treated as a fixed exogenous constraint for each scenario. There is no additional stochastic variability beyond that present in the historical data; consequently, we report exact counts of complex admissions, missed complex cases and false positives rather than confidence intervals from multiple replications. To check internal consistency, we confirmed that, for a given pair of thresholds, the numbers of complex admissions, complex-ward bed-days and misclassification counts produced by the simulation match those obtained by directly aggregating the underlying patient-level data and the confusion matrix of the predictive model. A formal prospective validation of the simulation-derived thresholds and REACH outputs in live clinical workflows is beyond the scope of this initial methodological study and is highlighted as an important direction for future work.

The **Stacked Ensemble A** model was simulated under varying capacities to determine cutoff values for assignment thresholds. This model integrates multiple ML techniques to predict patient complexity more accurately. By processing historical data, it identifies optimal thresholds that balance patient needs with available resources. In practice, patient assignment decisions are primarily driven by space availability and current ward occupancy. The simulation model explored the impact of different threshold values, facilitating precise identification of cutoff points crucial for patient assignment. These policy thresholds rank patients into groups based on their complexity probabilities, instrumental in effective triaging. By establishing thresholds, the simulation aims to reduce bed blocking and optimise resource utilisation within the complex ward.

The actual occupancy metrics and rates were excluded from REACH training due to variability in ward capacity over the study period and inconsistent recording in the dataset. This is a notable limitation but presents an opportunity for improvement by integrating only available ward capacity data in future research, allowing dynamic adjustments to thresholds based on actual bed availability. Policy thresholds were determined using historical data and various capacity scenarios of the complex ward, aiding informed patient allocation decisions based on current capacity. The simulation was conducted via data trace, limiting the ability to run multiple replications to quantify uncertainty. To enhance reliability, future simulations should include multiple iterations to provide a range of possible outcomes for decision-making. This aspect should be clearly communicated, acknowledging that the threshold settings are bespoke to the current operational realities of the OPR ward. Ultimately, the final REACH policy threshold based on patients' probabilities of being in the complex group can be demonstrated in Eq. (3).

3 $${t}_{REACH} = \left\{\begin{array}{lr}Green& {p}_{i} < 0.260\\Yellow& {0.261 < p}_{i} < 0.851\\Red & {p}_{i}> 0.852\end{array}\right.$$

A simulation over one year was conducted using data trace, with the initial complexity thresholds defined as Yellow for ${0.3<p}_{i}<0.8$, Red for ${p}_{i}>0.8$ and Green for values below 0.3. A total of 261,773 patient events were analysed: 167,019 (63.8%) assigned to Green, 4,173 (1.6%) to Red and 90,581 (34.6%) to Yellow. Among these, 183 (0.07%) were complex but not identified by REACH and 5,534 (2.11%) were not complex but predicted to be complex.

In order to avoid arbitrary cut-offs, we treated the REACH thresholds as policy parameters and carried out a systematic grid search over candidate lower and upper probability values for the Green, Yellow and Red groups. For each candidate combination, we ran a data-trace simulation over the one-year period and calculated three quantities: mean and maximum complex ward occupancy, the number of complex patients missed by REACH (false negatives) and the number of non-complex patients assigned to the complex ward (false positives). Thresholds that led to persistent occupancy above the available complex bed capacity were discarded. Among the remaining candidate settings, we examined the trade-off between false negatives and false positives and selected threshold values on this efficient frontier that reduced missed complex cases while keeping the additional load on the complex ward within realistic operational limits. The final thresholds in Eq. (3) therefore reflect an explicit balancing of safety (minimising missed complex patients) against resource use (avoiding excessive occupation of complex beds), rather than purely ad hoc values.

After refining the complexity thresholds as per Eq. (3), of the same 261,773 patients, 195,299 (74.6%) were classified as Green, 10,727 (4.1%) as Red and 55,747 (21.3%) as Yellow. In this improved classification, 131 (0.05%) were complex but not identified by REACH and 3,442 (1.32%) were not complex but predicted to be complex. Adjusting the thresholds improved the model's ability to distinguish complex from non-complex patients, reducing misclassified complex patients from 183 to 131 and false positives from 5,534 to 3,442.

**Fig. 6:**
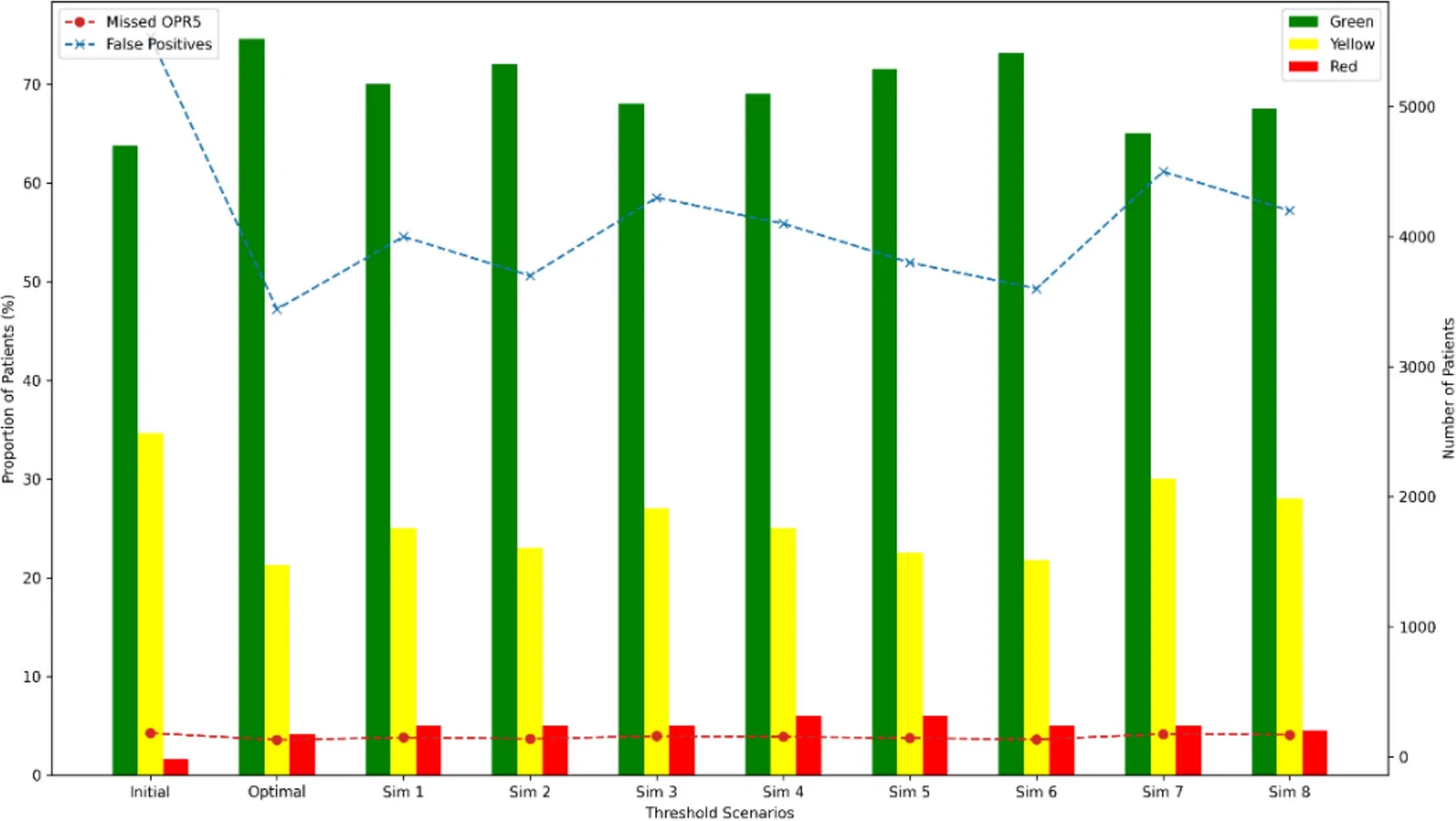
Comparison of reach complexity thresholds across simulated scenarios

As shown in Fig. 6, the proportion of patients assigned to the Green, Yellow and Red REACH categories varies systematically across the initial thresholds, the optimal thresholds and simulations Sim 1 to Sim 8. The dashed red line indicates complex patients missed by REACH (false negatives); the dashed blue line represents false positives. Initially, higher proportions of patients were classified as Yellow, with higher numbers of false positives and missed complex patients. Refining thresholds increased the proportion of Green patients and decreased Yellow, reducing false positives and missed complex patients, as shown by the downward trend of the blue and red dashed lines from the initial model to the optimal version.

During the simulation, some Yellow priority patients could not be admitted to the complex ward due to full occupancy. Even Red patients occasionally faced admission delays when the ward was full. Since actual ward occupancies were not available, future work incorporating dynamic capacity information will enable the algorithm to adjust thresholds and assignments based on bed availability, ensuring more responsive prioritisation. For instance, during low occupancy, thresholds could be adjusted to admit more Yellow and Green patients, maximising resource use and minimising waiting times. Conversely, during high occupancy, the algorithm could prioritise only the most critical cases (Red). Although there are currently no predefined bands within the groups, the thresholds were developed based on complexity probabilities. This means bands could be created within each group, including Red, to further prioritise patients based on their likelihood of requiring complex care. This dynamic adjustment will adapt to fluctuating patient volumes and resource availability, leading to more efficient healthcare delivery. Hospital resource utilisation is critically influenced by patient classification and prioritisation. The model's ability to predict patient complexity and adjust thresholds dynamically aims to optimise resource utilisation within the complex ward. Accurate categorisation helps prevent bed blocking, ensuring high-priority patients receive timely care. Efficient bed utilisation improves patient outcomes and enhances hospital operational efficiency, balancing patient needs with available resources and reducing under or over-utilisation of beds, which impacts care quality.

Furthermore, within each REACH group, patients can be prioritised based on their complexity probability $${p}_{i}$$. Table 4 presents an illustrative subset of the patient prioritisation stage for the first five patients in each REACH group. In this example, Table 4, patients in the Red group (complex threshold with $${p}_{i}>0.96)$$ exhibit the most significant challenges, characterised by a high number of comorbidities, length-of-stay and elevated scores on scales assessing clinical severity, such as PCCL, risk of decline (CHESS), care priority (MAPLE) and emergency department visit likelihood (DIVERT). This suggests a substantial need for resources and monitoring due to their severe health conditions and high risk of adverse outcomes. Patients in the Yellow and Green groups show progressively lower complexity probabilities and associated indicators. The Yellow group complexity probabilities range from approximately 0.813 to 0.846, indicating moderate needs. In contrast, the Green group, with probabilities as low as 0.102 to 0.139, represents the least complex cases, characterised by fewer comorbidities, shorter hospital stays and lower scores on the aforementioned scales. In this example, only the top five patients from each group are showcased. In the Yellow group, the patients showcased exhibit a non-negligible complexity probability, but the algorithm should undergo validation through feedback from clinicians and decision-makers for cases with lower priority.

**Table 4:** Patient prioritisation using complexity probabilities

Patient ID	Complexity Probability	Complexity Group	Number of Comorbidities^a^	Length of Stay^b^	PCCL^c^	CHESS^d^	MAPLE^e^	DIVERT^f^
P238	0.973	Red	5	80.5	4	5	5	4
P514	0.969	Red	3	55.1	3	4	5	6
P562	0.964	Red	6	54.9	3	4	5	4
P278	0.962	Red	5	30.9	3	3	3	5
P625	0.959	Red	3	17.8	4	3	5	5
P343	0.846	Yellow	4	30.6	3	4	4	5
P113	0.842	Yellow	2	15.8	3	4	3	6
P473	0.837	Yellow	3	10.2	3	3	5	6
P764	0.823	Yellow	3	7.3	3	3	5	2
P701	0.813	Yellow	2	9.5	2	3	4	4
P454	0.139	Green	2	5.4	2	3	4	3
P867	0.135	Green	1	4.3	2	3	3	2
P702	0.113	Green	2	30.6	3	1	2	2
P939	0.105	Green	1	22.0	2	2	4	3
P125	0.102	Green	1	6.7	0	2	4	1

**a**; Number of comorbidities extracted from interRAI assessment disease diagnosis list of primary and active diagnoses. **b**; Total length of hospital stay for the patient from admission to discharge for a specific episode. **c**; Patient clinical complexity level comes from the DRG program and the clinical severity within the record is identified. **d**; The Changes in Health, End-Stage Disease, Signs and Symptoms Scale (CHESS) was designed to identify individuals at risk of severe decline. It can serve as an outcome where the objective is to minimise problems related to the decline in function or as a pointer to identify persons with unstable conditions. Initially developed for nursing home residents, CHESS has been adapted for use with other instruments in the interRAI suite. It has a 6-point scale from 0 (not at all unstable) to 5 (highly unstable), with higher levels predictive of adverse outcomes such as mortality, hospitalisation, pain, caregiver stress and poor self-rated health. **e**; MAPLe differentiates people into five priority levels based on their risk of adverse outcomes. People in the lowest priority level have no major functional, cognitive, behavioural, or environmental problems and are considered self-reliant. The highest priority level is based on ADL impairment, cognitive impairment, wandering, behaviour problems and the InterRAI nursing home risk CAP. **f;** The Detection of Indicators and Vulnerabilities for Emergency Room Trips (DIVERT) Scale is a decision-support tool identifying a person’s likelihood of future unplanned emergency department (ED) visits. The DIVERT Scale ranges from 1 (lowest risk) to 6 (highest risk) for future ED visits

### Discussion and limitations

This research explored innovations in healthcare service design and delivery for acute older patients, providing insights for research, policy and practice. Hospital administrators should consider implementing older-persons-specific interventions to decrease hospital length-of-stay and costs, improve patient flow and reduce long-term care placements [17, 71, 72]. Decision-makers should recognise the economic and health benefits of these pathways, particularly with the ageing population. Although further research is required, interdisciplinary teams are clearly essential components of older-person-specific models. Clinicians should consider a multidisciplinary team of geriatricians, geriatric nurses, physiotherapists, occupational therapists, dieticians and social workers to improve care.

The Recognising Episodes of Acute Complexity in Health (REACH) for pathway assignment and prioritisation shows potential in addressing inefficiencies and maintaining patient throughput. Health policy measures should focus on specialised pathways for the frailest older patients, especially those admitted through ED with acute illnesses [17]. Policymakers should consider additional predictors for complex patient definitions between Māori and non-Māori populations [73, 74]. The research supports the use of patient prioritisation aids for transparent, fair resource allocation. Enhancing existing infrastructures for cost-effective data use and developing adaptable models are needed to accommodate varying data availability and institutional capabilities. Policymakers should streamline transitions to advanced decision support tools. Practical solutions for ML and decision support tools should be developed in collaboration with healthcare professionals to ensure real-world applicability and effectiveness. This collaborative approach will enhance the relevance and impact of ML tools in healthcare, leading to more informed, efficient and effective patient care.

This study represents one of the initial efforts to develop an older-person-specific predictive decision support tool that categorises patients into complex and non-complex pathways using their clinical and operational outcomes for prioritisation. Despite achieving its goals, limitations remain. In New Zealand, integrating AI into healthcare can play a crucial role in addressing health disparities and biases affecting communities like Māori and Pasifika. However, it is essential to combine AI with culturally sensitive approaches and community engagement to effectively combat the impacts of historical injustices. Engaging indigenous leaders and ensuring data privacy and trust are vital to promote minority participation, respect cultural diversity and prevent new inequalities.

While this study adhered to Māori data sovereignty principles, ensuring that Māori data was collected, stored and used with respect for Māori ownership and control, future efforts should go beyond these principles by actively involving Māori and other indigenous populations in the design and development of decision support tools. This means incorporating indigenous cultural values, preferences and priorities into the algorithms and processes, ensuring the tools align with the needs and aspirations of these communities. Such efforts would create more inclusive and culturally appropriate solutions that genuinely reflect and respect indigenous worldviews.

Despite the novelty of REACH, it has some computational limitations, including the absence of a feedback loop for algorithm outcomes and results based on expert judgments. This issue is common in static ML models that do not adapt after initial training, necessitating periodic EHR re-evaluation. A reinforcement learning (RL) approach could dynamically refine pathway allocation and prioritisation. Moreover, the thresholds of REACH, initially set from baseline data, could be enhanced through a feedback mechanism, allowing adjustments based on actual ward capacities, resource constraints and input from clinicians and decision-makers. This method will not only enhance the robustness of the algorithm but also build clinician trust in AI-based models by establishing a data-driven system that meets the usability criteria of decision-makers.

For the current study, the REACH Green-Yellow–Red thresholds were tuned using historical data and simulation-based occupancy analysis rather than formal clinical utility functions or consensus thresholds. This provides a transparent, reproducible statistical basis for the cut-offs, but it does not yet constitute full clinical validation of the decision boundaries. The thresholds should therefore be interpreted as ward-specific policy settings for the study period, rather than universally optimal values. In future work, we plan to refine these thresholds through co-design with geriatricians, emergency clinicians and stakeholders, and to evaluate candidate operating points prospectively in ‘silent run’ mode before deployment. This will allow explicit incorporation of risk tolerance for missed complex patients, acceptable additional workload on complex wards and equity considerations into the final threshold choices.

A further limitation is that some of the predictors used in the current AutoML pipeline, including length-of-stay, readmission and total cost, are not available at the moment of ED triage. As a result, the REACH model reported here should be interpreted as a retrospective proof-of-concept for complexity-aware prioritisation and capacity planning, rather than as a fully implemented real-time triage tool. A triage-ready deployment would require retraining REACH on a reduced feature set restricted to variables observable at or before ED presentation, followed by prospective “silent run” validation in clinical workflows. Designing and evaluating this streamlined, real-time version of REACH in partnership with clinicians, including equity-focused indicators for Māori and Pasifika patients, is an important focus for future work.

Another limitation of REACH is the potential bias within the predictor variable, specifically whether patients were admitted to a complex ward for older patients. Relying on this variable as the main predictor of complexity assumes clinicians' admission decisions are infallible, which may not be true. Hence, a data-driven model could be created where human input is only used for validation purposes to remove the biases within the model as much as possible. Future efforts should enhance models’ scalability and adaptability across various healthcare settings, especially in managing data flow in outpatient clinics. The goal is to develop flexible models requiring minimal data inputs, making them widely applicable and effective at improving patient prioritisation.

Additionally, while REACH has shown promise in patient prioritisation, it does not include ward occupancy as a variable, primarily because ward capacity was variable over the study period and not consistently recorded. However, one significant impact of this work has been the initiation of systematic ward capacity recording in hospital systems. This improvement ensures that capacity data will be available for future analyses, enabling the refinement of REACH to incorporate ward occupancy. This could allow the model to be revisited with capacity included as a key variable. The current policy thresholds for triaging patients in the OPR ward were determined using historical data and simulation under different capacity scenarios. These thresholds are tailored to the existing capacity of the OPR ward, ensuring more efficient and appropriate patient allocations. Future studies can extend REACH based on the capacity of the ward with changing thresholds. This enhancement would not only refine patient prioritisation but also improve overall hospital resource management.

## Conclusion

The results of this study demonstrate that REACH effectively streamlines patient pathway assignment and prioritisation for older patients with acute conditions. Because REACH is built on routinely collected EHR and interRAI data and existing ward configurations, it can be embedded into current admission, triage and bed-allocation workflows without requiring new data collections or radical service redesign. By integrating machine learning models, REACH assigns patients to appropriate complex or non-complex pathways and prioritises them based on their complexities, considering operational and clinical outcomes. This approach addresses key barriers to equitable healthcare delivery, offering practical solutions to optimise resource allocation in underserved and resource-limited settings. In doing so, it provides a concrete mechanism for health service planners and policymakers to operationalise equity and patient-flow objectives within older-persons’ services, rather than treating these goals as purely conceptual. The use of structured Big Data, specifically EHRs, significantly diverges from traditional methods such as clinical scores, highlighting a lack of similar studies and underscoring the utility of the proposed predictive model. From a practical perspective, the economic viability of advanced systems is crucial in the demanding, resource-limited contexts of healthcare settings, particularly in older persons’ care. The REACH framework is deliberately modular and data-driven, allowing local retraining of the AutoML model and recalibration of policy thresholds so that different hospitals, districts or countries can tailor the tool to their own demand, capacity and equity profiles.

However, there remains a need for flexible frameworks capable of handling varying data accessibilities and organisational strengths to extend the reach of analytics-based predictive tools. In particular, REACH model offers a novel framework to improve healthcare delivery for underserved populations, including older adults. Beyond offering direct operational benefits, the results underline broader structural challenges, such as limitations in resource capacity leading to patient overcrowding in geriatric care settings. This study introduces an innovative solution to a pressing healthcare issue, providing an effective decision-support tool with potential for national implementation in New Zealand and possibly internationally. Its reliance on widely used classification and assessment systems (e.g. ICD-10, AR-DRG and interRAI) further supports transferability to other jurisdictions that employ similar standards. The findings underscore the inefficiencies of traditional triage and prioritisation systems in meeting the needs of geriatric patients. Implementing REACH for older patient pathway assignment and prioritisation can lead to significant improvements in patient outcomes, reductions in wait times and overall hospital performance. Additionally, this model advances efforts to address health inequities by ensuring older adults receive timely and appropriate care, regardless of socioeconomic or geographic constraints.

Future policy-focused work could formally integrate REACH-derived complexity indicators and thresholds into geriatric service planning, performance targets and national guidance for acute older-persons’ care. This study not only contributes to the literature by providing a customised tool for acute older patient care but also lays the groundwork for future research to explore the integration of such predictive models in healthcare settings worldwide, supporting more equitable, efficient, and effective care for ageing and underserved populations.

## Supplementary Information

Below is the link to the electronic supplementary material.

Supplementary file1 (DOCX 549 KB)

## Data Availability

The data that support the findings of this study are available from the authors but restrictions apply to the availability of these data, which were used under license from the Te Whatu Ora Health New Zealand Waikato District for the current study, and so are not publicly available. Data are, however, available from the authors upon reasonable request and with permission from the Health & Disability Ethics Committees at the Te Whatu Ora Health New Zealand Waikato District, University of Waikato and University of Auckland.
