# Supplementary material for: Reach: Recognising episodes of acute complexity in health: a predictive older patient prioritisation machine learning model
Source: Health Care Manag Sci. 2026 Jul 29;29(3):37. doi: 10.1007/s10729-026-09783-5 (PMC13421209; doi:10.1007/s10729-026-09783-5)
Supplement: Supplementary file 1 — Supplementary file1 (DOCX 549 KB) [file 10729_2026_9783_MOESM1_ESM.docx]

**Appendix 1. MAthematical Presentation of REACH**

This section provides a mathematical representation of Recognising Episodes of Acute Complexity in Health (REACH). Although this study did not aim to find an optimal solution or solve the mathematical problem of patient prioritisation, the presented model offers a foundation for further research that can explore and address this challenge.

For each patient $pt$ in the set of patients $Pt$, REACH determines their pathway assignment by calculating the probability of complexity $p_{i}$, utilising a function that incorporates patient-specific features $F_{ik}$ and historical patient-specific data weights $w_{k}$. This probability not only determines the initial pathway assignment but also prioritises patient treatment within each category, ensuring that those with higher complexities are given precedence, subject to the availability of necessary resources. Accordingly, each pathway is characterised by a series of healthcare interventions, differentiated by the model as either requiring complex $C_{j}$ or non-complex $C_{j}^{'}$ resource allocations. REACH also accounts for the specific resources needed, including $a_{ij}$ for complex, and ${a'}_{ij}$ for non-complex pathways, by each patient $pt$, ensuring that the total resource consumption does not exceed the available capacity for each type of care. Hence, a set of decision variables can be defined as Eq. (A1).

| $x_{i}=\left\{ \begin{aligned} \begin{matrix} 1 & if patient i assigns to complex pathway \end{matrix} \\ \begin{matrix} 0 & if patient i assigns to non-complex pathway \end{matrix} \end{aligned} \right.$ | (A1) |
| --- | --- |
| $y_{i}=\left\{ \begin{aligned} \begin{matrix} 1 & if complex patient i is prioritised \end{matrix} \\ \begin{matrix} 0 & Otherwise \end{matrix} \end{aligned} \right.$ |  |
| $z_{i}=\left\{ \begin{aligned} \begin{matrix} 1 & if non-complex patient i is prioritised \end{matrix} \\ \begin{matrix} 0 & Otherwise \end{matrix} \end{aligned} \right.$ |  |

The prioritisation mechanism is governed by two primary objectives: (a) categorising patients into complex and non-complex pathways, and (b) prioritising patients in each pathway based on complexity probabilities. This dual-objective approach facilitates efficient resource utilisation and ensures that patients are treated in a manner that best matches their needs. Accordingly, based on the objective of the model, a mathematical model was designed to show the objective function along with the constraints of the model. A summary of the parameters within the model is presented, followed by the foundational model of REACH.

$\boldsymbol{p}_{\boldsymbol{i}}:$ The estimated probability of complexity.

$\boldsymbol{T}:$ The probability threshold to determine if patient $i$ is complex or non-complex. Determined based on the occupancy metrics from the simulation model.

$\boldsymbol{F}_{\boldsymbol{ik}}:$ The value of feature $k$ for patient $i$,e.g., age, length of stay, etc.

$\boldsymbol{w}_{\boldsymbol{k}}:$ Weight of feature $k$. It signifies the importance of the feature $k$ for each patient.

$\boldsymbol{b}:$ The bias term. $w_{k}$ and $b$ are determined using historical data/train data.

$\boldsymbol{C}_{\boldsymbol{j}}:$ The total available capacity of resource j needed for complex patients.

$\boldsymbol{C'}_{\boldsymbol{j}}:$ The total available capacity of resource $j$ needed for non-complex patients.

$\boldsymbol{a}_{\boldsymbol{ij}}:$ The amount of the resource $C_{j}$ required by complex patient $i$.

$\boldsymbol{a'}_{\boldsymbol{ij}}:$ The amount of the resource ${C'}_{j}$ required by non-complex patient $i$.

$\boldsymbol{M}\left( \boldsymbol{.} \right)$: The threshold showing the relationship between estimated probability $p_{i}$, and the decision variable $x_{i}$ ensuring consistency in the classification based on the threshold$T$.

$\boldsymbol{ml}\left( \boldsymbol{.} \right):$ The machine learning function used to estimate $p_{i}$.

| $\min\sum_{i} x_{i}$ | (A2) |
| --- | --- |
| $\max\sum_{i} p_{i}y_{i}+\sum_{i} p_{i}z_{i}$ | (A3) |
| $Subject to$ |  |
| $p_{i}-T\leq M\left( x_{i} \right),\forall i$ | (A4) |
| $y_{i}\leq x_{i}\leq{1-z}_{i},\forall i$ | (A5) |
| $\sum_{i} a_{ij}y_{i}\leq C_{j}, \forall j$ | (A6) |
| $\sum_{i} {a'}_{ij}z_{i}\leq{C^{'}}_{j}, \forall j$ | (A7) |
| $p_{i}=ml(F_{ik}w_{k}b) , \forall i$ | (A8) |
| $x_{i},y_{i},z_{i}\in\{0,1\}$ | (A9) |

The objective function Eq. (A2) along with constraint Eq. (A4) aims to categorise patients into complex and non-complex pathways based on the $p_{i}$ value. Accordingly, if $p_{i}$ is greater than the threshold, then the model assigns patient $i$ to complex pathway, and if the threshold is below 1, then the model assigns patient $i$ to non-complex pathway. Moreover, the objective function Eq. (A3) prioritise the patients in each pathway. This is necessary due to the limited availability of resources for patients in each pathway. Therefore, more complex patients are prioritised if enough resources are available. Constraints Eq. (A5) keeps $y_{i}$ and $z_{i}$ as free variables for complex and non-complex patients, respectively. Furthermore, constraints Eq. (A6) and Eq. (A7) are resource constraints, in which, patients in each pathway are prioritised until none of the resource constraints are violated. This constraint can be customised based on the number of beds, rooms, and resources. It should be noted that, non-complex patients are also getting prioritised within this model, and if decision-makers decide to exclude them from the model, $z_{i}$ can be removed. Ultimately, Eq. (A8) calculates the complexity probability for each patient using the given weights from the historical data. This probability is calculated from the ML pipeline associated with the model.

Moreover, the next step is to incorporate the concept of virtual streaming, where resources are logically segregated and allocated across prioritised patient streams within a department, which can enhance system performance through more effective partitioning of capacity. Since this model is only presented to showcase the presentation of REACH design and was not initially developed to be solved and extract an optimum solution from the mathematical model, a streamlined approach is utilised in order to introduce the concept of virtual streaming to the model by adjusting the approach with focusing on the allocation of resources and prioritisation without significantly altering the core structure of the existing model. Accordingly, instead of explicitly defining virtual streams through variables, we introduce a dynamic partitioning mechanism within the constraints that manage the allocation of $C_{j}$ and ${C^{'}}_{j}$, the total available capacities for complex (Eq. (A10)) and non-complex (Eq. (A11)) patients, respectively.

| $\sum_{i} a_{ij}y_{i}\leq{\alpha_{j}C}_{j}, \forall j$ | (A10) |
| --- | --- |
| $\sum_{i} {a'}_{ij}z_{i}\leq{\left( {1-\alpha}_{j} \right)C^{'}}_{j}, \forall j$ | (A11) |

In which, $\alpha_{j}$ represents the dynamic partitioning coefficient for resource $j$, determining the proportion of $C_{j}$ allocated to the highest priority patients within the complex pathway. This coefficient can be adjusted based on the current demand, priorities, or specific departmental policies, effectively implementing the concept of virtual streaming by dynamically allocating resources between different priority groups. Ultimately, it should be noted that while the mathematical model presented here was designed explicitly for REACH, this research intends not to employ this model directly to solve or optimise the mathematical problem. Instead, our primary aim was to present this model in a mathematical format to delineate the underlying principles and mechanisms that will drive the model. By doing so, we aim to provide a clear and structured mathematical representation of the concepts that inform our ML approach, ensuring the transition from a theoretical model to a practical computational framework is transparent and robust. Subsequently, the initial model presented in Eq. (A2) to Eq. (A9) can be expanded with the AutoML pipeline considering the CASH problem based on the following modifications.

| $\min\sum_{i} x_{i}$ | (A12) |
| --- | --- |
| $\max\sum_{i} p_{i}y_{i}+\sum_{i} p_{i}z_{i}$ | (A13) |
| $Subject to$ |  |
| $p_{i}-T\leq M\left( x_{i} \right),\forall i$ | (A14) |
| $y_{i}\leq x_{i}\leq{1-z}_{i},\forall i$ | (A15) |
| $\sum_{i} a_{ij}y_{i}\leq{\alpha_{j}C}_{j}, \forall j$ | (A16) |
| $\sum_{i} {a'}_{ij}z_{i}\leq{\left( {1-\alpha}_{j} \right)C^{'}}_{j}, \forall j$ | (A17) |
| $p_{i}=ml\left( A^{*},\lambda^{*},F_{ik} \right) whereA^{*},\lambda^{*}=arg\min_{A^{j}\in A,\lambda\in\Lambda} \frac{1}{k}\sum_{i=1}^{k} L\left( A_{\lambda}^{j},D_{train}^{i},D_{test}^{i} \right), \forall i$ | (A18) |
| $x_{i},y_{i},z_{i}\in\{0,1\}$ | (A19) |

Where $A^{*}$ and $\lambda^{*}$ represent the best algorithm and its hyperparameters selected through the AutoML process. This process involves running an optimisation loop over a set of algorithms $A$, and their hyperparameter spaces $\Lambda$ to find the combination that minimises the average loss $L$ across $k$-fold cross-validation on training $D_{train}^{i}$ and testing $D_{test}^{i}$ datasets. This process directly impacts how $p_{i}$ the probability of a patient being assigned to a complex pathway, is calculated, ensuring the decision is based on the most effective model identified by the AutoML process. This approach allows the model to dynamically adapt to the best ML strategy for the classification phase, enhancing the model’s accuracy and reliability in assigning phases.

Moreover, for each patient $x$, if $ml\left( A^{*},\lambda^{*},F_{ik} \right)>t$, then $x$ is considered complex and eligible for the complex pathway, in which $t$ is the complexity threshold within the initial model. Suppose there are $X$ patients within our historical data over a period of $D$days. An occupancy vector of length $D$ with a value of 0 can be initialised to calculate the occupancy vector of the complex ward. The threshold was set by evaluating the effect of changing occupancy (more details on setting up the threshold is defined in Section 3.5). It should also be noted that actual complex patients not predicted to be in complex wards by the model should also be monitored, and how many non-complex patients get sent to complex the ward. There is a trade-off here – the cost of the extra patients versus missing some patients who need to go there. Considering the varying capacities (25, 50, 75 and 109 beds), this simulation can be modelled as when for each $x$ in $X$, if $ml\left( A^{*},\lambda^{*},F_{ik} \right)>t$ then $occupancy[j, j+l] = occupancy[j, j+l] + 1$ otherwise is non-complex, where $j$ is the arrival day of the patient $x$, and $l$ is the length of stay of patient $x$. In Other words, Patients who met the defined complex threshold were identified and assigned to the complex pathway and for each such patient, the occupancy from their day of admission $j$through $j+l$ (where $l$is the length-of-stay) was increased by one. This method not only tracked the physical presence of patients but also highlighted the resource implications of managing complex cases.

Accordingly, the Stacked Ensemble A model was simulated through the varying capacities (25, 50, 75 and 109 beds) in order to extract the cutoff values for REACH threshold. Figure A1 illustrates the model's accuracy with different cutoff values to extract thresholds for REACH. It should be noted that Figure A1 was created under the scenario where the complex ward has a maximum capacity of 109 beds, and occupancy of the patients based on length of hospital stay was calculated to extract cutoff values. The occupancy simulation based on length of hospital for over 1 year was illustrated in Figure A2.


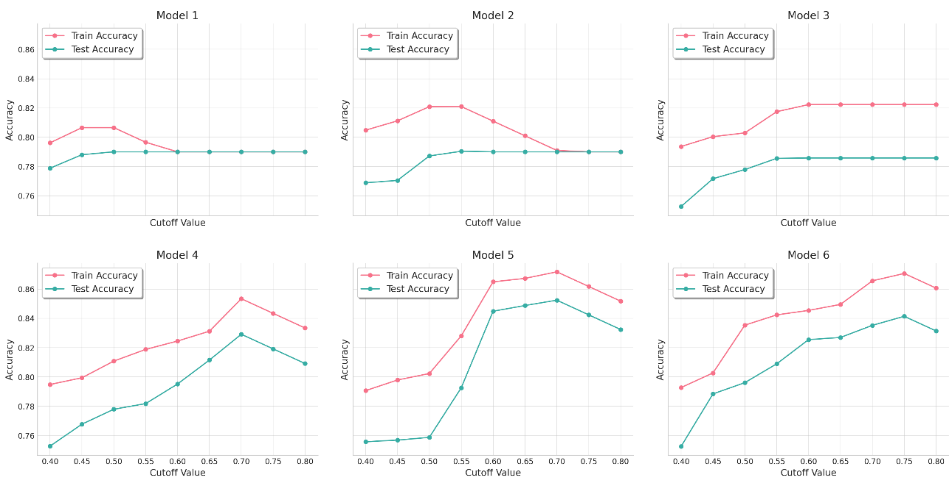


**Figure A1:** Evaluating cutoff values based on accuracy for the REACH complexity threshold.

Each curve corresponds to a candidate model from the AutoML pipeline simulated under the maximum complex-ward capacity scenario (109 beds). The x-axis shows the candidate complexity-probability cutoff, and the y-axis shows the corresponding classification accuracy on the held-out test set. For most models, accuracy rises to a peak and then declines as the cutoff is raised further; Stacked Ensemble A (the model retained for the final REACH pipeline) exhibits the highest peak accuracy and is used to extract the Green/Yellow/Red thresholds reported in Eq. 3 of the main manuscript.


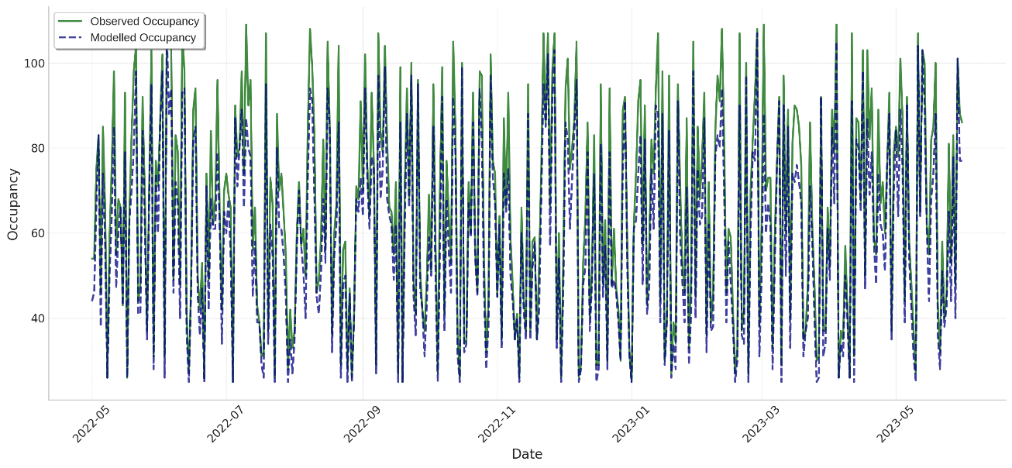


**Figure A2:** Simulating complex-ward occupancy and length-of-stay over a 12-month period.

The solid line shows observed daily occupancy of the complex Older Persons and Rehabilitation (OPR) ward; the dashed line shows modelled occupancy under the REACH allocation policy with thresholds derived from Stacked Ensemble A. Observed occupancy fluctuates between approximately 23 and 109 beds; modelled occupancy varies within the same envelope but reduces peak-day bed pressure relative to the historical baseline. The figure supports the choice of the operational capacity envelope used in the threshold-tuning grid search (minimum 21 beds, maximum 109 beds). The observed occupancy is shown in Figure A2, fluctuating between 23 and 109, while the modelled occupancy varies within the same range but distinctively differs from the observed data. This shows that the complex ward's minimum occupancy needs to be 21 beds with a maximum of 109 beds. Moreover, for most models in Figure A1, the accuracy decreases after reaching a peak, except for Model 3, where the accuracy stabilises post-peak. While only a snapshot of the models was illustrated in this figure, all models stabilised post-peak or decreased accuracy after a specific cut-off value.
